# What is the State of Organisational Compassion‐Based Interventions Targeting to Improve Health Professionals' Well‐Being? Results of a Systematic Review

**DOI:** 10.1111/jan.16484

**Published:** 2024-10-07

**Authors:** Camilla Littau Nielsen, Christina Louise Lindhardt, Lui Näslund‐Koch, Tove Faber Frandsen, Jane Clemensen, Connie Timmermann

**Affiliations:** ^1^ Hans Christian Andersen Children's Hospital Odense University Hospital Odense Denmark; ^2^ Department of Clinical Research University of Southern Denmark Odense Denmark; ^3^ Centre for Compassion in Healthcare, Department of Clinical Research, Department of Regional Health Research University of Southern Denmark Odense Denmark; ^4^ Centre for Research in Patient Communication Odense University Hospital Odense Denmark; ^5^ Faculty of Health Deakin University Burwood Australia; ^6^ Lægerne Postparken Kastrup Capital Region of Denmark Denmark; ^7^ Department of Design and Communication University of Southern Denmark Odense Denmark; ^8^ Centre for Innovative Medical Technology, Odense University Hospital University of Southern Denmark Odense Denmark

**Keywords:** burnout, care, compassion, occupational health, staff development, systematic reviews and meta‐analyses, work organisation

## Abstract

**Aim:**

To identify and assess the state of knowledge regarding compassion‐based interventions and outcomes, targeted to the organisational level, that aim to improve health professionals' well‐being.

**Design:**

Systematic review.

**Data Sources:**

Using the PICO model, the clinical question and search strategy were structured. The searches were performed on 20 September 2022 and 26 December 2023 in the Scopus, CINAHL, EMBASE, PsycINFO and ProQuest Dissertations & Theses Global databases. Content analysis was applied to analyse data, and the PRISMA and SWiM guidelines were followed for reporting.

**Results:**

Thirty‐eight studies, mostly from the United Kingdom and the United States, met the inclusion criteria and were quality assessed and analysed. Compassion‐based interventions that target the organisational level are quite new, thus representing a burgeoning initiative. In this review, many included quantitative studies revealed significant methodological challenges in effectively measuring organisational compassion (interpersonal relationships, organisational culture and retention/turnover). However, the review findings overall indicate that interpersonal connections between colleagues that foster a sense of community, through shared experiences, mindfulness and (self‐)compassion practices and social activities, may be a protective factor for well‐being. Further, the review emphasises the crucial role of management support in catalysing organisational changes to improve health professionals' well‐being.

**Conclusion:**

Evidence strongly suggests that fostering human interconnectedness among health professionals is associated with enhanced well‐being. Further rigorous studies are needed to validate these findings, clarify the organisational cultural aspects of compassion and develop an effective outcome measurement tool for organisational compassion.

**Practice Implications:**

Organisational compassion‐based interventions may help foster a culture of compassion within organisations, enhance health professionals' capacity for compassion and benefit both their well‐being and the quality of care provided to patients and relatives.

**Patient Contributions:**

This review is part of a larger project about compassion and includes two patient representatives (mothers of children with cancer) in the research team.

**Summary**

- This review summarises the existing body of knowledge and highlights knowledge gaps regarding organisational compassion which aim is to improve health professionals' well‐being.
- Fostering human interconnectedness among health professionals may increase their well‐being, thus improving health professionals' compassion capabilities in their work with patients and relatives.
- Organisational compassion outcomes are identified with relevance for the development of an effective measurement tool for compassion in healthcare organisations.

## 1 Introduction

Healthcare systems around the world operate under substantial pressure due to factors such as increasing demand, an ageing population and the prevalence of chronic diseases (Bardhan, Chen, and Karahanna 2020; Osareme et al. 2024). Consequently, health professionals (HPs) may face increasing demands in their increasingly complex work (Bardhan, Chen, and Karahanna 2020), heightening the risk of stress and burnout (Zhang et al. 2018). Burnout constitutes a serious global concern, especially evident among HPs during the 2019 coronavirus disease (COVID‐19) pandemic (Ghahramani et al. 2021; Zhang et al. 2018). Such challenges affect the universal care covenant between human beings—the core of nursing—and influence the nature of care being delivered in our healthcare systems (Iles 2014).

In healthcare, compassion is receiving increasing attention and is commonly understood as a response to one's own or others' suffering that derives from a desire to help, followed by action to alleviate the suffering (Perez‐Bret, Altisent, and Rocafort 2016). Self‐compassion revolves around one's own experiences, while compassion expands the focus to encompass the experiences of others (Feldman and Kuyken 2011; García‐Campayo et al. 2024). Central to this process is mindfulness, which fosters mindful presence and embraces our vulnerability, shared humanity and concern for ourselves and others (Germer and Neff 2019). Mutual vulnerability enables us to initiate a movement towards one another that offers reciprocal support. This can help build human interconnectedness, which is understood as ‘being mutually dependent on one another for comfort and well‐being’ (Dong 2020, 39). In relationship‐based care, compassion has proven to be rewarding for both care receivers and care providers, revealing significant psychological and physiological effects on well‐being. This includes improved patient quality of life and better resilience and health outcomes in patients and HPs (Trzeciak, Mazzarelli, and Booker 2019). Resilience is ‘the process and outcome of successfully adapting to difficult or challenging life experiences, especially through mental, emotional and behavioural flexibility and adjustment to external and internal demands’ (American Psychological Association 2022).

The need for increased compassion in healthcare has been recognised for many years. The 2013 ‘Francis Report’ from Mid Staffordshire, UK, highlighted severe deficiencies in patient care, particularly the lack of compassion, which led to significant patient suffering. A cultural shift towards more compassion was deemed essential (Francis 2013). Similarly, a study conducted in Sweden in 2015, focusing on patients' experiences with the healthcare system 5 years after a bus accident, also emphasised the need and importance of compassion (Doohan and Saveman 2015). The lack of compassion from HPs was the pain most former patients recalled and found most burdensome after the physical injuries (Doohan and Saveman 2015). Subsequently, two American physicians spent years reviewing research literature, which, under the concept of ‘compassionomics,’ consolidates evidence showing that compassion and self‐compassion make a positive difference for patients, HPs and the healthcare system as a whole (Trzeciak, Mazzarelli, and Booker 2019). The concern that HPs are often unfairly blamed for a lack of compassion, results in interventions, such as self‐compassion practices, that aim to improve compassionate care by enhancing HPs' internal resources (Sinclair et al. 2017). However, the path forward may not necessitate an inward but rather an outward focus, empowering HPs to fulfil their inherent desire to alleviate the suffering of others (Sinclair et al. 2017).

To date, most research on compassion targeting HPs, including the growing number of literature reviews, has primarily focused on individual‐level solutions (Cohen et al. 2023; Lee and Cha 2023; Melnick, Sinsky, and Shanafelt 2023). The fact that HPs' well‐being is a shared concern between the individual and the organisation and entails shared responsibility to investigate, address, and prevent its root causes can thus be overlooked (Lown et al. 2024; Melnick, Sinsky, and Shanafelt 2023). The present study hypothesised that nurturing HPs' well‐being through compassion is a complex process involving both organisational, interpersonal and individual factors, and is an emerging initiative within healthcare organisations.

## 2 The Review

### 2.1 Aims

This systematic review aimed to assess the effects of compassion‐based interventions and outcomes, targeted to the organisational level, that aim to improve health professionals' well‐being. Based on this knowledge, the study sought to establish an initial foundational framework for fostering compassion in healthcare organisations that benefit health professionals.

### 2.2 Design

Cochrane's Handbook for Systematic Reviews of Interventions (Chandler et al. 2019) was applied in preparing and conducting this systematic review. The Preferred Reporting Items for Systematic Reviews and Meta‐Analysis (PRISMA) guideline (Page et al. 2021) and the Synthesis Without Meta‐analysis (SWiM) guideline (Campbell et al. 2020) were followed for reporting. Details about the review can be found in the protocol registered in the International Prospective Register of Systematic Reviews (PROSPERO; Page, Shamseer, and Tricco 2018) with ID: CRD42022374641.

### 2.3 Search Methods

The review's overall question was: ‘What does it take to become a compassionate healthcare organisation, fostering well‐being among HPs?’ This led to the specific focus on identifying factors for organisational compassion that benefits HPs, including the establishment of a framework. The PICO model was used to structure the clinical question and facilitate the search (Frandsen et al. 2020). It was structured as follows: P = HPs with patient contact; I = compassion‐based interventions; C = any comparison; and O = organisational compassion. While all authors discussed and agreed on the clinical question, C.L.N. and T.F.F. were responsible for conducting an initial, unstructured search in the Scopus database on the clinical topic, to identify potential keywords. Subsequently, on 20 September 2022—and again on 26 December 2023—the structured search was performed by C.L.N. in the Scopus, CINAHL, EMBASE (Ovid), PsycINFO (Ovid) and ProQuest Dissertations & Theses Global databases, using both text words and thesaurus terms. Spelling variations and singular/plural were considered, and the search was limited to include studies written in English, Danish, Norwegian or Swedish. An example of the search string is provided in Table 1, while the full list is presented in Supporting Information 1.

**TABLE 1:** Search string example.

Database	Search string
Scopus	**S1)** TITLE‐ABS‐KEY ( ( ( compassion* OR kindness OR empath* OR sympath* OR genero ) W/3 ( unit* OR principle* OR department* OR organisation* OR communit* OR practice* OR workplace* OR culture* OR environment* OR facilit* OR philosoph* OR organization* OR climate* ) ) AND ( hospi* OR health* ) ) **S2)** TITLE‐ABS‐KEY ( ( compassion* OR kindness OR empath* OR sympath* OR genero* ) W/3 ( hospi* OR health* ) ) **S3)** ( TITLE‐ABS‐KEY ( ( compassion* OR kindness OR empath* OR sympath* OR genero* ) W/3 ( hospi* OR health* ) ) ) OR ( TITLE‐ABS‐KEY ( ( ( compassion* OR kindness OR empath* OR sympath* OR genero ) W/3 ( unit* OR principle* OR department* OR organisation* OR communit* OR practice* OR workplace* OR culture* OR environment* OR facilit* OR philosoph* OR organization* OR climate* ) ) AND ( hospi* OR health* ) ) )

*Note*: * indicates that all types of endings are included in the search.

### 2.4 Eligibility Criteria

Any published study that evaluated a compassion‐based intervention on organisational compassion outcomes in hospitals, hospices and general practices was relevant to this review. This included books and theses, but excluded reviews, conference abstracts and research protocols. Given that compassion is about alleviating suffering (Perez‐Bret, Altisent, and Rocafort 2016), interventions had to reflect such an effort, intending to improve HP's well‐being. This encompassed interventions directly referring to compassion, those referring to elements of compassion, such as mindfulness and resilience, and those referring to concepts linked to compassion like empathy, sympathy, generosity and kindness. Further, we included interventions that aimed to increase knowledge, skills and human care and connection, supporting emotion regulation, based on our shared humanity (Feldman and Kuyken 2011; Germer and Neff 2019). These factors, in addition to shared values, beliefs, norms, practices, leaders' behaviour, interpersonal relationships (Mascaro et al. 2020) and retention and commitment to the workplace (Beardsmore and McSherry 2017), comprised eligible organisational outcomes. Hence, outcomes for HPs' well‐being overall included the aspects of interpersonal relationships, job satisfaction, workplace commitment, values, turnover, retention and culture.

### 2.5 Study Selection

Using the Covidence software, eligible studies derived from the bibliographic databases were merged, and duplicates were manually removed by C.L.N. Studies were then double‐screened based on (1) title/abstract and (2) full text, utilising a developed screening tool. The screening process involved the authors C.L.N., C.L.L., L.N.‐K. and C.T., with C.L.N. responsible for screening all studies, and C.L.L., L.N.‐K. and C.T. sharing the full number. Disagreements (conflicts) were resolved by a third of the screening authors, C.L.L., L.N.‐K. or C.T. who were not involved in the particular conflict with C.L.N., and involved fourth author (T.F.F.) arbitration, if necessary.

### 2.6 Search Outcomes

**FIGURE 1:**
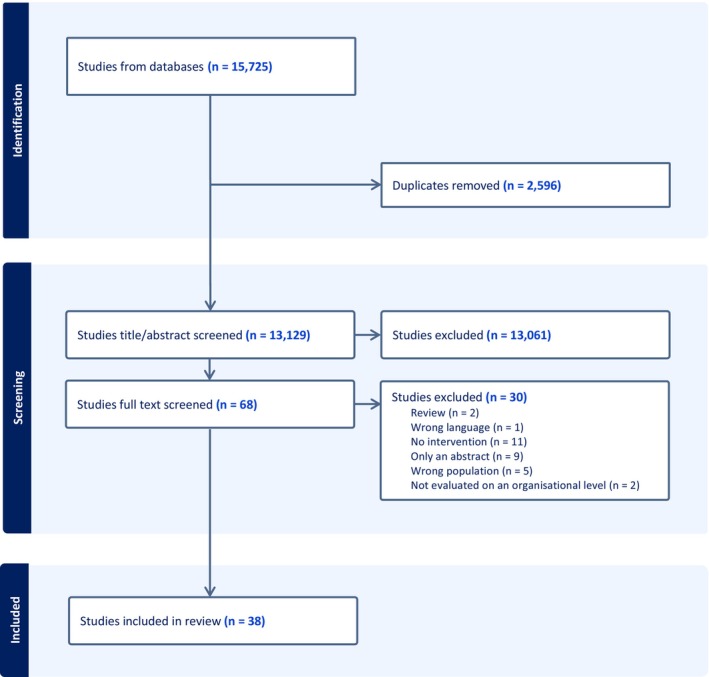
Study selection flowchart.

Among 15,725 studies assessed for eligibility, 2596 were removed as duplicates. The remaining 13,129 studies were title/abstract screened, of which 13,061 were excluded for not meeting the inclusion criteria. Thus, 68 studies were full‐text screened, which resulted in the inclusion of 38 studies. One study appeared twice, as an article and part of a thesis, and is presented as one (MacArthur 2014; MacArthur et al. 2017). The full screening process and reasons for exclusion are outlined in Figure 1 (Page et al. 2021).

The 38 included studies were published within the period 2008–2023, including most in the years 2020–2023 (*n* = 20) and from the United States (*n* = 15) and the United Kingdom (*n* = 10; Table 2). The studies either involved HPs in a nonspecified sense (*n* = 12) or specific (interdisciplinary) cohorts. These mostly consisted of nurses (*n* = 17), physicians (*n* = 11), certified nursing assistants (*n* = 6), psychologists or therapists (*n* = 3), and/or midwives (*n* = 3), across a wide range of specialisms, primarily in hospitals. The way in which each intervention relates to compassion is highlighted in Table 2, using italic text. Many refer explicitly to compassion by developing (self‐)compassion interventions, including mindfulness and resilience practices (Table 2). Others typically focus on emotion regulation and building resilience through increasing HPs' knowledge (education), establishing safe spaces for sharing and getting to know each other, and/or promoting human care and connection in the workplace. An overview of designs for evaluating interventions is provided in Table 3 and detailed in Table 2. In total, they show qualitative (*n* = 23) and quantitative (*n* = 16) methods, primarily using interviews and surveys respectively. Finally, a wide range of outcome measures appeared, which are listed in Table 4 and further described in Table 2.

**TABLE 2:** Study characteristics.

First author, publication year and location	Aims	Interventions, including participating health professionals (HPs) and settings	Methods	Main findings
de Senneville et al. (2022), Australia	To investigate the views of junior doctors on adding kindness to handover by having an emotional support person at the labour ward handover.	The implemented ISBAR (Introduction, Situation, Background, Assessment and Recommendation) tool was added *kindness* (KISBAR) and used during labour ward handovers among 41 obstetric junior doctors affiliated with a hospital department. A staff member was incorporated as an emotional support person whose role was to support the team communication during handovers.	A qualitative ethnographic methodology was used. After around a year of implementation, two nonstructured hospital‐based focus group interviews of 30 min duration each, with 14 and 7 junior doctors, were conducted by a doctor not taking part in the intervention. Thematic analysis of the verbatim transcribed data was undertaken independently by two researchers.	Junior doctors who felt stress and uncertainty in labour ward handovers with colleagues experienced improved safety and a more supportive environment. Further, they experienced acknowledgment from the hospital regarding the importance of junior doctors' well‐being.
Nissim et al. (2019), Canada	To elucidate the subjective experience of receiving compassion, presence and resilience training, and perceptions of its benefits, risks or challenges.	The *compassion, presence and resilience training curriculum* included 30 nurses, physicians and other professionals from two interdisciplinary oncology teams at a large hospital in Toronto, Ontario, Canada. It unfolded over 8 weeks in sessions that lasted 1.5 h per week and was facilitated by a psychiatrist with extensive mindfulness training. This curriculum included *workplace, mindfulness tasks (micro‐practices), education on cultivating presence and experiential instruction on mindful communication skills*, using in‐session dyadic exercises.	Within the period 1–5 months postintervention, qualitative, individual interviews were performed with 10 (out of 30 possible) participants from the initial two participating teams. Two researchers who did not take part in the intervention facilitation carried out interviews, lasting 30–90 min. Thematic analysis strategy was used to code the verbatim transcribed data, done by one researcher and checked by another.	HPs experienced acknowledgment and support from the hospital regarding self‐care as a shared responsibility of the individual and the organisation, which enhanced their motivation and engagement. The ‘simple’ micro‐practices enabled better interactions with colleagues. However, it was difficult to exhibit their vulnerability within the intervention teams, including daily colleagues, due to the feeling of jeopardising professional relationships and trust because of the ‘stiff’ organisational culture. Over time, HPs felt more safe and protected and opened up.
Copeland (2021), USA	To examine the feasibility, acceptability and effectiveness of brief, 5‐min interventions on […] perceived teamwork.	Twenty‐two full or part‐time nurses and one nurse aide working any shift at a suburban Level 1 trauma centre were randomly assigned into intervention or control groups. In the intervention group, nurses engaged in one or more of 4–5‐min activities during 6 weeks: *(1) Meditation, (2) Outside breaks, (3) Gratitude (thank and complement people) and (4) Journaling*. All were documenting their efforts.	A quasi‐experimental pilot study using pre–post surveys […] 21 items from the Agency for Healthcare Research and Quality Teamwork Perceptions Questionnaire). As 3 dropped out, 20 nurses completed pre–post surveys.	A significant difference in pre–post intervention communication score was observed for the journaling group (from a mean score of 8.5 (SD = 0.58) to 6.25 (SD = 1.7), *p* = 0.037). No other significant differences appeared for any of the groups (meditation, outside, gratitude, journal and control) with regard to communication and job satisfaction scores.
Schorch et al. (2021), USA	To implement and evaluate a wellness programme, providing interventions to decrease burnout and secondary traumatic stress and increase compassion satisfaction.	The wellness programme involved a postanaesthesia care unit (20 nurses, 5 patient care assistants, one assistant nurse manager and one manager) and lasted 16 months. It included three interventions: *(1) education, (2) social support and (3) healthy behaviours/self‐care*. Social events were planned outside work once a month, a private Facebook social media page was created, a ‘co‐worker of the week’ initiative was implemented and praise cards were available for *recognition purposes*.	A cohort study design, using self‐made pre–post surveys, including open‐ended questions. Fourteen (out of 27 possible) HPs completed the presurvey, while nine completed the post survey.	Postintervention, there were increases in co‐worker support, co‐worker recognition, teamwork morale and job satisfaction. Only teamwork morale increased significantly; from 3 (sometimes; medium) to 4 (often; high) on a 5‐point Likert scale, SD = 0.50.
Warriner, Hunter, and Dymond (2016), UK	To support staff to […] improve the culture of the organisation as a whole.	The 8‐week *mindfulness meditation course ‘Finding Peace in a Frantic World’* was offered to 46 staff members, affiliated with the Oxford University Hospitals NHS Foundation Trust, of whom 43 fulfilled. They were from the hospital (30%), community (30%), research midwives (9%), maternity support workers (18%), student midwives (9%), doctors (2%) and lecturers (2%). They attended an average of 87 (range, 50–100) % of sessions and were told to do 30 min of daily home practice for 6 days of the week each week.	Postintervention, participants (*n* = 34; response rate = 79%) completed an unspecified survey. Four to 6 months postintervention, they (*n* = 23; response rate = 53%) completed a follow‐up survey.	Participants reported at 4–6 months follow‐up that the course had a positive impact on their work–life (*n* = 21, 91%) and the culture of their workplace (*n* = 13, 59%), with the majority reporting that they have used their learned skills either daily or weekly.
Flanders et al. (2020), USA	To evaluate the impact of a resilience programme for paediatric health professionals on […] employee engagement and nursing turnover	The programme, involving 150 paediatric intensive care nurses at a children's hospital in the south‐central part of the United States, was implemented in 2017. It included education about *compassion fatigue, burnout, compassion satisfaction and resilience*. Resilience strategies included formal (twice a year, led by a trained paediatric ethicist) and informal (once a month with a chaplain) debriefings, art, music and pet therapy. They were trained in skilled communication, effective decision‐making, true collaboration, meaningful recognition, appropriate staffing and authentic leadership. Finally, a Facebook page was established and monthly physical activities.	A cohort study design using pre–post surveys. Moreover, data on nursing turnover and employee engagement were obtained. The 6 Press Ganey employee engagement items were calculated in 2016 before the intervention started, while professional quality of life scores were measured postintervention during the first 3 months of 2018. Data on nursing turnover from 2016 were compared with those of 2017.	Nursing turnover scores increased by 6%, while employee engagement scores increased from a mean score of 4.15 (*n* = 82) to 4.18 (*n* = 75), although they were not statistically significant (*p* = 0.22 and *p* = 0.67, respectively).
Cosentino et al. (2021), Italy	To evaluate the effect of the expressive writing protocol on the levels of […] organisational commitment, as well as to evaluate the perception of the usefulness of expressive writing protocol.	The intervention involved 50 HPs (nurses, social and health workers, auxiliaries, doctors and psychologists) within palliative care hospital units or services in northern and central Italy. It was carried out over 6 months (August 2019 to February 2020) and was undertaken in two steps: (1) In both groups, the writing mandate, lasting 20 min, was given. (2) During 3 weeks, 3 home‐based additional sessions in expressive *writing on emotions, sensations and thoughts* or neutral writing, 3–4 days apart, were performed, followed by a meeting.	A prospective experimental study with two groups; expressive writing (intervention group; *n* = 22) and neutral writing (control group; *n* = 28), using pre–post surveys with a time span of 3 weeks. These comprised […] the Italian version of the Organisational Commitment Questionnaire and ad hoc (self‐made) questionnaire evaluating the usefulness of the writing sessions.	Organisational commitment was evaluated based on three components; affective, continuance and normative commitment. Continuance commitment significantly increased in the intervention group (*Z* = −3.357, *p* = 0.001).
Duncan et al. (2011), USA	To describe the design, rationale, use and perceived impact of the walk‐in Restore & Renew Wellness Clinic pilot project.	Within a calm, healing environment with trained personal, all employees at the Restore & Renew Wellness Clinic at a United States Department of Defence hospital, including physicians (11%), clinical supervisors (12%), nurses (25%) and medical technicians, were guided to bring *full awareness to their surroundings and into states of being fully present in the moment, using mindfulness*. They were guided to let go of stress, enhance re‐integration of previously dissociated sensations or emotions and bring balance to the activation/collapse dynamics, as well as offered ear acupuncture, clinical acupressure and zero balancing (osteopathic medicine).	A prospective cohort study using a 1‐page self‐made survey, including space for free‐text comments, after each visit.	Among repeat visitors, the proportion reporting strong agreement with perceived long‐term benefits increased by the number of visits on ease in relationships with co‐workers.
Knudsen et al. (2021), Denmark	To explore experiences of the impact of an 8‐week mindfulness‐based stress reduction course on their clinical practice, including encounters with colleagues […].	The *mindfulness‐based stress reduction course* for 6 nurses and doctors affiliated with a Danish cardiology hospital department unfolded during November 2018. This course encompassed the *cultivation of mindfulness through meditation, body scan and yoga exercises* for 2.5 h once a week over 8 weeks, as well as a day of 8 h in silence. Moreover, home training of 45–60 min duration per day was encouraged.	Using interpretative phenomenological methods, 6 qualitative, individual interviews were performed by a researcher other than the trained mindfulness teacher facilitating the course.	Promotion of acceptance and understanding of colleagues from other professions, increasing the awareness of one another's vulnerability and challenges, which resulted in feelings of interpersonal connection.
Adamson et al. (2018), Canada	To examine the perceived impact of an arts‐based narrative training intervention.	The once‐weekly 90‐min sessions of the intervention, guided by an experienced facilitator, involved 8 nurses at an urban Canadian paediatric rehabilitation hospital. Sessions included quiet reading, expressive writings and discussions about the themes: *‘The Other Side of Care’, ‘Building Perspective’, ‘Obstacles to Empathy’, ‘Limits to Rehabilitation’, ‘Making Room for Hope’ and ‘A Letter to Myself’*.	A qualitative study, using 1‐h semi‐structured, pre–post intervention interviews with a time span of around 8 weeks.	Nurses experienced a more cohesive sense of community with colleagues, interrupting negativity and fostering interpersonal compassion, which was in contrast to prior. Furthermore, the nurses' abilities to turn to each other for professional reflection, as well as to share vulnerability and work‐related suffering increased after the intervention.
Burnet et al. (2023), USA	[…] to assess the impact of a weekly wellness programme for surgical residents.	From March 2017 to June 2018, surgical residents were encouraged to attend 1‐h weekly in‐person breakfast, mindful conferences (protected time) led by a skilled senior surgical facility mentor. The conferences included *creating a safe space, mindful meditation*, topic or speaker presentation and roundtable discussion.	A cohort study using validated tools measuring self‐reported mindfulness, self‐compassion, flourishing and burnout postintervention.	Free text feedback provided in the comment field in the surveys showed that nearly all respondents appreciated having protected time for reflection and discussion. The programme further symbolised that their managers cared about their well‐being.
Landry et al. (2018), Canada	To share the hospital's experience of designing and implementing campaigns for promoting kindness.	Activities for HPs (and patients) at Bluewater Health community hospital in Sarina and Petrolia, Canada undertaken since January 2017 included leaders' ‘walk the talk’ and leaders encouraging HPs to take a *time‐out or talk something through*. A group of HPs discussed 4 principles for creating a *culture of kindness* and generated activities. Committed to a random act of kindness each day, they influenced their colleagues to find ways to incorporate kindness. *Acknowledgment* of colleagues in general and those going ‘the extra mile’, and *building community* through innovative and creative efforts was in focus.	Pre–post engagement surveys (in 2016 and 2018) were undertaken by a third party.	The perceived ‘culture of kindness’ increased (from 61.9% to 69.9%). The same applied to the questions ‘hospital as a place to work’ (from 69.8% to 76.3%) and ‘can trust this organisation’ (increased 4.2%). The culture was perceived to change positively; people were smiling and had eye contact to a greater extent. Likewise, more people were greeting each other.
Drenkard (2008), USA	To improve nurse satisfaction and retention by decreasing work intensity, streamlining cumbersome nursing processes and creating a human caring environment in the workplace.	Nurses (*n* = 621) affiliated with Inova Health System in northern Virginia, comprising four medical units (intervention group) and four surgical units (comparison group) across four hospitals participated in the intervention, which was carried out in three phases. (1) Nurses in the intervention group *worked together in teams*, and the work intensity in the departments was lowered, providing more time around patients for the nurses. (2) Caring processes were implemented through a theory‐based practice model. (3) The *human caring work environment* was developed through user‐involvement and implemented.	A quasi‐experimental, between‐subjects, naturalistic, longitudinal study, using pre–post measures on nurse satisfaction (Healthcare Environment survey) […], as well as data on nurse turnover from the National Database for Nursing Quality Indicators data set. Moreover, nurses engaged in focus group interviews.	With regard to nurse satisfaction (response rate; 37%–52% [pre] and 50% [post]), improvements appeared statistically significant (*p* = ≤ 0.05) for the Healthcare Environment score (change score = 0.09) and subscales for the relationships with co‐workers (0.21), and the workload (0.21). Not significant improvements involved relationships with physicians, pride in the organisation, promotional opportunities, being present and improved relationships with peers. Comparisons of nurse turnover and vacancy data revealed decreasing (not significant) trends. Qualitative feedback revealed that nurses felt able to care for each other as co‐workers and had support from co‐workers and leaders to spend more time with patients, fostering job satisfaction.
Rushton et al. (2021), USA	[…] Secondary outcomes related to an experiential educational curriculum included changes in […] work engagement and turnover intention.	*The programme (Mindful ethical practice and resilience academy)*, involving frontline nurses from two hospitals in a large academic medical system, unfolded within the period 2016–2018. It included 6 experiential sessions; 24 h of face‐to‐face, interactive training based on educational and evaluative methods. Five sessions incorporated didactic experiential practices, role play, video review *mindfulness* and group activities; 1 session involved high‐fidelity simulation with trained actors and a facilitated reflective debriefing.	A longitudinal pre–post intervention design was utilised, comparing intervention and (informal) control group outcomes.	In total 192 nurses completed pre–post intervention surveys, while this counted 223 in the comparison group. The sessions were attended by 94%, and 88% completed the simulation session. The work engagement increased significantly from a mean score of 4.97 (SD = 0.95) to 5.28 (SD = 0.82), *p* = 0.001, while turnover intentions decreased; however, not statistically significant (2.75 [1.44]–2.48 [1.41], *p* = 0.05).
Flowers et al. (2018), UK	To evaluate the ‘taking care, giving care’ rounds to further improve the concept.	The 1‐h group‐based rounds, unfolding within natural work settings in the Aneurin Bevan University Health Board, were led by a facilitator and host. *Entering a positive frame of mind, discussions on values, self‐compassion and compassion within the workplace*, using clear questions, took place in pairs and within larger groups, in which everyone was expected to contribute.	Participants affiliated with medical departments, acute psychiatric departments and community services provided 62 recorded statements. A representative sample of comments was presented.	Paying compliments to each other was good for the team morale. Further, it was appreciated to take time out to discuss team values and how to approach practice and show compassion. Discussing team approach provided feelings of being valued as a member of the team.
Vanstone et al. (2020), USA/Canada	To examine the organisational effects of the 3 Wishes Project.	The 3 Wishes Project is an end‐of‐life intervention in which clinicians elicit and implement wishes that honour the dying patient and comfort family members through *addressing suffering*. 72 clinicians were enrolled.	Applying a pragmatist epistemological approach, descriptive data were generated and undertaken in a directed content analysis.	Acts of compassion were incorporated into clinical work. Clinicians were aware of their colleagues' suffering and responded compassionately. The colleague connections were strengthened, which enhanced the meaning and purpose of their work.
Stacey et al. (2020), UK	To explore the experience, acceptability and feasibility of Resilience Based Clinical Supervision to support transition to practice in newly qualified nurses.	*Resilience Based Clinical Supervision* was implemented in 6 Healthcare Trusts across the East Midlands (UK) and facilitated by registered HPs (predominantly clinical educators). This included discussions on group conditions needed *to create a safe space, mindfulness exercises, commitment to cultivating and maintaining a compassionate flow* and consideration of the role of the internal critic. Over a 1‐year period, 266 newly qualified nurses received a minimum of six 2‐h sessions.	Eight focus group interviews with a total of 42 newly qualified nurses who recently completed 6 sessions were conducted and transcribed verbatim. A deductive approach to data analysis, focusing on perceived outcomes, challenges, experiences and the best practice related to Resilience Based Clinical Supervision, was applied independently by 6 project team members.	The experience of increased courage to act and ask for help, as well as address negative working conditions, e.g. with managers, was reported. Peer support occurred within the group, which was perceived as a distinctive and psychologically safe space where their feelings were validated in a nonjudgemental way, promoting well‐being. A sense of belonging arose among peers, which provided them with feelings of not being alone with their current feelings.
Curtis et al. (2017), UK	To develop a sustainable programme of ‘compassion awareness training’ that engaged with diverse colleagues within the partner organisations across the region, building on existing compassion initiatives.	HPs within 4 National Health Service organisations in the South East of England (two large hospitals; a mental health service provider and a community‐based primary care service provider) participated. An *online toolkit of compassion resources*, including digital stories, group activities (*sharing random acts of kindness, mindfulness exercises and compassion indicator statements*) and links to literature. A train‐the‐trainer approach was adopted, and the project team members facilitated training days and provided monthly follow‐up meetings.	Data were generated through focus group interviews and individual interviews during the training days and monthly follow up meetings to explore views, feelings, values and experiences. Thematic analysis was used to analyse the audio‐recorded and transcribed data.	HPs experienced that toolkits could help them recognise and celebrate compassion within the team. The toolkits and group activities increased awareness of the responsibility to recognise compassionate acts of others. Digital stories served as an easy and quick way to initiate a conversation about compassion. The compassion indicators were useful for pointing up existing good practices and generating critical dialogue within teams, also focusing on emotional labour, self‐compassion and peer support.
Small, Feldman, and Oldfield (2017), USA	To better understand the Narrative Medicine programme's impact and for the purposes of quality improvements in care among HPs.	The interprofessional programme in Narrative Medicine (AfterWards) at the Children's Centre of the John Hopkins Hospital was held monthly on a voluntary drop‐in basis. Every session consisted of three parts: (1) discussing literature or art with a medical theme, (2) *writing about experiences based on a prompt and (3) sharing thoughts and reflections*.	Eighteen months after the intervention, 14 health professionals (3 residents, 3 social workers, 4 attending physicians, a child life specialist, a fellow and 2 nurses), with an average of participation in three (range; 1–9) sessions, participated in individual, qualitative interviews lasting on average 31 min.	AfterWards provided an interdisciplinary community in the hospital setting where interprofessional HPs could meet and talk about personal and professional experiences. This, and discussions on art, literature, etc., made them relate to each other to a greater extent and connect in valuable ways. HPs also felt that AfterWards overturned the usual hierarchy that predicted how people interact at work and provided insight into colleagues' roles in the team.
Goodrich (2012), UK	To assess whether the Schwartz Centre Rounds could transfer from the United States to the United Kingdom, and whether it would achieve a similar positive impact on […] teams and hospital culture.	The 1‐h rounds were open to all staff members at two NHS trust hospitals providing acute care in the west of England and Central London. From October 2019 to October 2020, the initial 10 rounds were carried out, involving 1250 staff members across the two hospitals. A panel of a facilitating doctor and one or two other staff members were presenting their cases for 10–15 min, in which *they described their role, issues and emotions that arose due to the issues. Afterward, other staff members could ask questions, reflect on and share experiences related to the topics*.	Qualitative face‐to‐face or telephone interviews were performed initially after 1–3 rounds (*n* = 18) and at the end of the intervention after at least 10 rounds (*n* = 23). The 18 participants were organising committee members (*n* = 14), panellists (*n* = 2) or facilitators (*n* = 2). The 23 participants were organising committee members (*n* = 11), panellists (*n* = 4) or facilitators (*n* = 4). Thirteen were interviewed twice. Familiarisation/thematic frameworks were used for data analysis.	The rounds were experienced to help increase the respect, empathy and understanding between staff members, particularly beneficial for staff members in junior positions. A greater appreciation of how colleagues feel about their work had a greater potential for interprofessional work and promoted collaborations between teams and individuals. The hospital environment was reported as less hierarchical as colleagues could meet and share as equals. The symbolic gesture from the management in valuing the staff and their well‐being was powerful, and rounds were seen as an instrument to build and share values on an organisational level.
Sands, Stanley, and Charon (2008), USA	To test the feasibility and effectiveness of providing narrative training for the purpose of promoting […] team building.	The 60‐min weekly sessions were open to all employees at the paediatric oncology inpatient unit at the Morgan Stanley Children's Hospital in New York. Each session began with 10 min of silent writing and read aloud what had been written. Then *the discussion related to each writing began*, *inviting other members to share their thoughts/feelings*. This was facilitated by 2 skilled faculty members.	A mixed method cohort study. Besides pre–post surveys, the 19 employees (6 physicians, 12 nurses and one psychosocial member) participated in a 1‐h focus group interview held by a member of educational research within the last week of the intervention.	Focus group discussions revealed that the responsiveness and sharing process from the sessions were transferred into their interpersonal interactions during work, and they felt that they knew their colleagues even better (personally and professionally).
Shah, Lambrecht, and O'Callaghan (2017), New Zealand	To evaluate the efficacy of staff support reflective rounds to discuss complex emotional and psychosocial issues that arise in caring for patients and families.	The rounds involved 276 HPs (average attendance of 23 participants per round) affiliated with Auckland City Hospital. Rounds unfolded monthly from April to September 2014 and were facilitated by a trained senior psychologist. A panel of 3 staff members had 5 min each to present a case, in which they described *their role, issues, and emotions that arose due to the issues. Afterward, other staff members could ask questions, reflect on, and share experiences*.	Evaluations (*n* = 218) of 12 rounds were collected and presented to the Directorate of Cancer and Blood Services.	Improved understanding of colleagues, including their approaches and perspectives, was reported, providing a shared experience and being more open to their colleagues. HPs could mirror themselves in the challenges of colleagues, and they acknowledged the need for being kind to colleagues. Moreover, 87% reported working better with colleagues, while 97% reported gaining insight into how others think and feel in their work with patients.
Dreher (2018), USA	To explore the effect of the implementation of a compassion fatigue awareness and self‐care skills educational training on […] retention.	The intervention included 45 (of 47 possible) certified nursing assistants at a non‐for‐profit, state‐run veterans nursing home operated by the state's Department of Mental Health in the Southeast region of the United States. From September 18 to September 22, 2017, 14 sessions of a 90‐min workplace educational programme on *compassion fatigue awareness and self‐care skills in physical, mental and emotional domains* were offered, and all should attend one.	The study used a quasi‐experimental, independent group pre–post test design using surveys and data on retention. Ten participants dropped out during the 1‐ and 3‐month data collection period postintervention.	In the 3‐month period following intervention, the retention rates increased, while the utilisation of supplemental agency staff decreased. From September 23, 2017 to December 23, 2017, there was no voluntary turnover. Monthly retention rates for 2017 were compared to monthly retention rates from 2016. All revealed improvements in retention; October (from 52% to 95%), November (from 44% to 86%) and December (44% to 84%).
Allen et al. (2020), UK	To evaluate the experiences of the Schwartz Rounds.	The Schwartz Rounds were open to 150 mental HPs within an inpatient acute psychiatric unit at Derbyshire Healthcare NHS Foundation Trust. Of those, 93 (62%) attended at least one round, of whom 33 (35%) attended more than one. Forty (43%) nurses, 12 (13%) medical professionals and 11 (12%) occupational therapists participated. The 1‐h rounds were held once a month and included a multidisciplinary team to *discuss, listen, process and understand any emotional distress, and reflect on personal experiences of distress and ways of coping*.	A mixed‐methods study using […] qualitative focus group interviews. Nine (of the 33 possible invited participants) participated in a focus group interview at 1‐month follow‐up, done by a researcher unknown to the participants. The interview lasted 40 min and was transcribed and analysed by an author (codes checked by another author), using thematic analysis.	HPs found it highly valuable to listen to and mirror feelings with colleagues, which enhanced colleague empathy. Likewise, having this space, in which it was acceptable to share emotions, was valued. However, some felt that the duration of the rounds was too short to disclose their emotions, while others felt smaller groups on the team level would make it easier to find confidence to speak (build trust). Here, some also conflicted with the perceived expectation to hide emotions from patients and colleagues to be professional. Most reported that Rounds had not been in place long enough to have an impact on clinical practice.
Dewar and Cook (2014), UK	To support staff to work together to develop a culture of inquiry that would enhance the delivery of compassionate care.	The 12‐month leadership programme, using the principles of *appreciative relationship centred leadership*, involved 86 nurses (2 associate nursing directors, 5 clinical nurse managers, 23 charge nurses/ward managers, 23 senior staff/registered nurses and 33 staff/registered nurses), of whom 10% were part of the current nursing force within an acute hospital in a rural part of Scotland.	A study using mixed methods in a formative way, which means that findings along the intervention were fed back into the programme. Data were generated using initial staff culture questionnaire (*n* = 86), including staff responses from participating areas (*n* = 319), ongoing reflections, staff‐reported developments, case studies and 65 staff interviews (answers noted).	The staff culture conveyed security and continuity, yet feelings of accomplishment and significance were low. HPs felt well‐informed and understood expectations but perceived limited influence over processes and challenges in discussing tough issues. Many became more aware of their leadership strengths and contributions, feeling less judgmental. Enhanced relationships were noted due to increased sensitivity to others' perspectives, fostering openness and trust. HPs improved their communication skills, reframed behaviours and used peers as supportive and critical friends.
Maben et al. (2021), UK	To uncover and make explicit the explanations for how, why and for whom Schwartz Rounds work.	Schwartz Rounds are largely used in the United States and United Kingdom and encompass sourcing stories and panellists, crafting and rehearsing stories in panel preparation, and the 1‐h round itself, including presenting the case for the panel of up to four staff members in front of the audience. Two trained facilitators (senior physician and psychosocial practitioner) supported and steered the discussion of emerging themes, allowing space for the audience to reflect and/or comment on similar *experiences/feelings after the panel of different HPs had described the impact the case had on them regarding the difficult, satisfying or demanding aspects of the emotional situation*.	A qualitative study, using realist evaluation methodology. A total of 48 facilitators and clinical leads in 45 settings in UK running Rounds in 2015 were interviewed by telephone. Nine case studies from sampled nationally and included interviews with clinical leads, facilitators, panellists and members of steering groups, audiences organisation boards and nonattenders (*n* = 177). Of these 225, 97 key informant interviews were analysed.	The Rounds created a sense of (psychological) safety for exhibiting vulnerability, which established trust and containment to share. Rounds allowed a break from the hectic, fast‐paced, protocol‐driven and hierarchical culture of healthcare work, providing a space of silence and stillness. This space for peace and reflection resulted in staff being more present. Likewise, a safe space for colleague dialogue was created, providing a community of commitment. Seeing others' vulnerability provided understanding of colleagues' pressure and behaviours, leading to greater empathy, tolerance and generosity, which led to more trust, compassion and kindness; wider cultural changes (‘ripple’ effect).
MacArthur (2014) and MacArthur et al. (2017), UK	To critically analyse the impact of the Leadership in Compassionate Care Programme and offer a conceptual model of factors that can embed compassionate care in contemporary healthcare environments.	The 3‐year study (2008–2011) included patients, relatives, NHS staff, lecturers and student nurses (a total of 106 participants) from 33 clinical settings in 8 wards. Four senior nurses and a lead nurse delivered the intervention to the staff in each ward and unit for 7–9 months, *involving emotional touchpoints eliciting individual stories of emotional experience, beliefs and values clarification to develop a common shared purpose/vision and understanding, and photo elicitation, discussing the meaning of compassionate care*.	A longitudinal qualitative study design using realistic evaluation methodology, through semi‐structured interviews (individual [*n* = 39] and focus group [*n* = 3] lasting from 57 to 120 min), informal observations of clinical practice, attendance at programme meetings/conferences and review of research outputs from the programme team. In 4 wards, data were collected over 3 years, while in another 4 wards over 2 years, and analysed using thematic analysis.	It became evident that being treated compassionately was not only important for patients but also for the staff. The focus was centred on ‘relationships with all people that you meet’; hearing the voices of patients, relatives and staff. The staff felt increased confidence to open work discussions, and on a team level, a strong emphasis on communication and staff support became embedded in the high‐adopting wards. They developed ‘catch‐up meetings’, ‘caring conversations’ and team discussions, as well as weekly reflections with the hospital chaplain.
McManus and Robinson (2022), USA	To illuminate the experiences of […] HPs participating in Compassion Rounds.	*Compassion Rounds intent to bring physicians, chaplains and other HPs together with patients and their families in conversations that solely address emotional, psychological and spiritual needs during difficult times by focusing on patients and relatives' coping with the experience of illness and hospitalisation*. Compassion Rounds were performed in a neonatal intensive care unit.	A qualitative study involving 15 HPs (8 chaplains, 3 physicians and 4 other nonspecified HPs) participated in individual interviews from October 2018 to June 2019. They had participated in an average of 43 Rounds. More than half of them agreed to participant in focus group interviews. A thematic analysis method was applied.	Besides increasing their feeling of connection with the families, HPs felt more connected to each other, fostering better relationships.
Orellana‐Rios et al. (2018), Germany	To explore the feasibility and effectiveness of ‘on the job’ programme.	The 10‐week intervention included 28 HPs from all work areas of a palliative care centre in a faith‐based community hospital in Bonn, Germany, and was led by a skilled meditation teacher. Intervention included *(1) mindful presence, (2) cultivation of loving‐kindness, (3) the practice of Tong‐len and (4) integrating those practices into daily work activities*. A total of 57% attended ad least 6–9 practice days and the initial introductory session, with a compliance rate of 70%.	A pilot study with a mixed methods approach, using […] pre–post surveys, encompassing […] work situation and goal attainment scaling, home‐written journals (*n* = 20) and postintervention interviews with all participants, ranging from 27 to 90 (SD = 52) minutes. Integrative interview analysis was used for analysing data.	HPs' level of work enjoyment increased significantly (from a mean score of 7.75 (SD = 1.44) to 8.19 (SD = 1.66), *p* = 0.005). Their levels of work satisfaction (7.75 [1.34]–8.19 [1.47], *p* = 0.117) and enforcement (7.83 [1.81]–8.15 [1.46], *p* = 0.274) increased nonsignificantly. HPs felt helped in cooperating mindful pauses into work and strengthened in interpersonal connecting skills and supported in relationship building. They also experienced better team communication and conflict management.
Schrøder et al. (2022), Denmark	To evaluate a formalised peer support programme, called ‘the Buddy Study’, to emotionally support each other after adverse events.	The intervention encompassed (1) a compulsory 2‐h seminar for 5–15 participants, (2) self‐selection of two Buddies (*colleagues as sup‐porters in case of adverse/traumatic events during shifts*) and (3) a *buddy activation and response system* within 4 weeks after an event. HPs (*n* = 250) at two departments; Emergency (physicians) and Obstetrics/Gynaecology (midwives and nursing assistants) at Odense University Hospital, Denmark, participated.	A mixed methods study, using cross‐sectional pre‐(*n* = 191)‐post (*n* = 167; response rate = 67%)‐surveys comprised of two close‐ended questionnaires, in addition to two open‐ended questions and three individual interviews.	Most HPs reported that the seminar provided mutual insight and understanding and the programme had increased attentiveness to one another and others' well‐being and a sense of feeling safe. Yet only a third felt that the programme had led to more openness after adverse events. The programme encouraged an open and compassionate culture where peer support was seen as valuable. One challenge was that natural peer support already was in use.
Trygg Lycke, Airosa, and Lundh (2023), Sweden	To explore nurses' and assistant nurses' experiences of the project to increase empathy and compassion through mindfulness delivered via workshop and smartphone app.	A total of 51 (of 200 eligible) nurses and assistant nurses affiliated with an emergency department in a major metropolitan area took part in the intervention undertaken in December 2016 to May 2017. The intervention consisted of a 3‐h workshop that focused on *compassion, empathy, mindfulness and silent lunch*. Participants were given access to a smartphone app for *meditation exercises such as breathing, loving‐kindness, resting and body scan*. They were encouraged to practice mindfulness daily for 8 weeks.	A qualitative study using the phenomenological research method of systematic text condensation. Eight individual interviews with 5 nurses and 3 assistant nurses were performed.	By increasing their self‐awareness (being present in the moment and centring one‐self), HPs increased their awareness of colleagues (and patients) making them reflect on their own and colleagues' attitudes and behaviours. Behavioural change was observed, involving greater eye contact with colleagues (and patients), and greeting colleagues in the corridors. The workshop provided space for sharing, widening one's horizon to recognise that others feel the same way and learning to see things from others' perspectives.
Drobinska (2020), UK	To explore the feasibility and acceptability of a compassion‐focused group therapy for staff.	Within two NHS inpatient sites in North Wales, two groups of HPs (nurses, assistant psychologists, healthcare assistants and activity coordinators, *n* = 12) participated in 6 weekly 2‐h sessions facilitated in the day‐time by two clinical psychologists trained in Compassion‐Focused Therapy. They had an introduction to *compassion and psychoeducation regarding the human brain and emotion‐regulation systems. The sessions encompassed experiential compassion cultivation practices*, e.g., *soothing rhythm breathing and compassionate imagery*. Four (33%) completed 4–6 sessions.	A mixed quantitative and qualitative methods design was employed, using self‐made surveys to assess HPs views after each session (*n* = 10). Eight semi‐structured interviews were conducted after the group to explore their immediate experience of the group. Those who attended less than 4 sessions were interviewed to explore reasons for attrition. Interview transcripts were analysed using thematic analysis.	The session provided possibilities to meet and share experiences at and to give and receive compassion. The experience of getting together with colleagues and making them realise that they are not alone in their struggles and that others are feeling the same (shared humanity) was valuable. However, being busy and short‐staffed provided guilt of attending sessions while colleagues were running faster in the clinic (the main reason for attrition). They reported having a culture of taking care of others and not themselves and they felt little value and recognition for their work and efforts. A lack of management support as the needs of the ward were prioritised over staff well‐being was also reported.
Henderson et al. (2022), Australia	To explore the concept ‘capital’ through the study of successive interventions and outcomes in a quaternary intensive care unit.	Around 180–190 nurses at a metropolitan public adult teaching hospital (Level 1 Trauma Centre) in Australia participated in unit‐based activities, led by leaders. Activities encompassed *‘Compassion Cafés’, feedback sessions about patient progress, open forums with medical staff about decision‐making, creative approaches to rostering and an internal‐external peer support system*.	A cohort study using validated workplace surveys (and care delivery measures) at four time points (2015, 2016, 2017 and 2019) over 5 years (2015–2019). The nurses' survey response rates were between 39%–79%, with 35%–45% consistently responding.	Over the years, the overall perception of workplace culture remained consistently satisfactory or good, showing no significant changes across subscales from 2015 to 2016 or 2017. However, notable differences emerged in 2019, with statistically significant lower median scores for all factors.
Courtenay (2016), USA	To improve […] employee engagement results.	Open to all, clinical and nonclinical staff at Castle Medical Centre were invited to join the Empathy Enhancement Programme lasting from March to June 2015. A total of 983 (of 1054 employees) attended from 72 different departments (93% participation). Staff on a leave of absence were excluded (7% of total staff). The programme included an initial intervention of a workshop, *including education, relaxation exercises and role‐plays*, among others.	The programme was evaluated by attendance at the empathy enhancement workshop based on […] attendee evaluation of the workshop shortly after the intervention (July 2015), pre (2014)‐post (September 2015) Gallup Q12 employee engagement surveys (94% employee participation) and employee turnover rate.	With regard to employee engagement, the overall score was at the 51st percentile ranking in Gallup's Hospital Level Database in 2014, which increased to the 78th percentile postintervention. In 2015, there was an increase in total turnover of all staff from 19.6% to 21.3%. Of full‐time and part‐time staff, there was an increase from 13.9% to 15.7%. Full‐time and part‐time registered nurse turnover went from 15.7% to 19.5%.
Knudsen et al. (2023), Denmark	To explore HPs' first‐hand experiences of how attending a mindfulness stress reduction course influenced their work life, including their relationships with […] colleagues and how they integrated mindfulness into practice.	A total of 56 (nurses, midwives, physicians and a healthcare assistant), counting 25% of all eligible staff members, from a cardiology department and an obstetrics and gynaecology department in Denmark participated in the course. The course consisted of 8 weekly 2.5‐h group sessions and a full day session, facilitated by a certified mindfulness instructor. The course focused on teaching HPs to *observe thoughts, sensations and feelings in a nonjudgemental way, and to help them exercise (meditation, yoga and body scan)*. Further, the course included joint reflections. HPs were encouraged to practice mindfulness for 45 min per day.	A qualitative study using multiple methods, involving observations of 3 physicians, 3 nurses 1 midwife, 15 individual interviews and 6 focus group interviews with 6–8 per group. Thematic analysis was applied for analysing data.	Changing behaviour among HPs and how this had the potential to foster a more compassionate culture were discussed. Enhancing the ability to stay connected to oneself increased the awareness of positive communication with colleagues. This included awareness of not passing on stress to colleagues to avoid a stressful atmosphere. Further, HPs felt better at not adopting colleagues' stressful behaviours. The course also created a safe space to connect, providing common understanding and awareness of each other, which strengthened relationships with colleagues. However, since only a small part of staff participated, a common understanding was lacking in the organisation.
Kratzke et al. (2023), USA	To evaluate the impact on well‐being of a self‐compassion programme modified for surgical residents.	*The self‐compassion programme ‘Resilience Training for the Healthcare Community’ (RTHC*) was delivered in multiple sessions over 6 weeks (a total of 6 h of instruction) by course instructors (certified teachers of Mindful Self‐compassion). Surgical residents from a single academic institution were invited to participate during the spring of 2018, 2019 and 2020. The RTHC involved *practicing self‐compassion, ‘deeply living’ and self‐compassion for caregivers*.	A cohort study evaluated through validated tools measuring anxiety, burnout, stress and patient health, in addition to yearly focus group discussions that constituted a formative evaluation, leading to adjustment along the intervention.	Discussions showed it was great that leaders started to recognise and address the problem of HP burnout and take the lead to encourage the residents to nurture own well‐being by task prioritisation (protected time from duties to attend RTHC). Yet, some reported RTHC to conflict with the ‘surgical residency culture’ and personality of surgeons. During the first year of RTHC, residents did not feel safe to disclose their vulnerability (being open) with their chiefs. This changed due to the creation of a nurturing environment by the course leaders, which symbolised care, making residents appreciate the opportunity for self‐care.
Franco et al. (2024), USA	To illuminate how the Self‐Compassion for Healthcare Communities (SCHC) helped participants develop a compassionate way of relating to themselves and how they implemented self‐compassion practices in their everyday lives.	The SCHC programme included *informal and brief exercises, including paying attention to the soles of one's feet during emotionally burdensome patient situations, attaching a badge card to one's lanyard guiding mindfulness and self‐compassion exercises, as well as phrases and breathing exercises to apply during interactions with others in the workplace*. SCHC was carried out over 6 weeks and facilitated by two Mindful Self‐Compassion teachers. Every week, participants met for 1‐h during lunch, and the structure of these meetings was: opening discussion, the topic of the day introduction, a guided practice, small and large group discussions and closing.	A qualitative study using semi‐structured interviews with 9 (social workers and nurses) of 23 invited participants from the SCHC programme. Qualitative content analysis was applied to analyse data.	Social connecting via the SCHC programme improved interpersonal interactions and helped develop shared humanity (recognising not being alone with difficulties) and better relationships with colleagues (and patients). This broadened the sense of community in the workplace and helped employ self‐compassion in clinical practice. It was easier to share emotions with and understand colleagues, including articulating their own needs as well as identifying colleagues' needs, but also setting boundaries toward each other. Further, participants reported using each other as partners, e.g. reflecting with each other on behaviours and helping each other create breaks, which helped to maintain productivity.

*Note*: Italic text indicates how each intervention is related to compassion.

**TABLE 3:** Quality assessment of included studies.

Cohort design	Validity of results							Results			Generalisability	
	Q1	Q2	Q3	Q4	Q5	Q6	Q7	Q8	Q9	Q10	Q11	Q12
Henderson et al. (2022)												
Burnet et al. (2023)												
Shah, Lambrecht, and O'Callaghan (2017)												
Duncan et al. (2011)												
Schorch et al. (2021)												
Copeland (2021)												
Orellana‐Rios et al. (2018)												
Landry et al. (2018)												
Flanders et al. (2020)												
Warriner, Hunter, and Dymond (2016)												
Dreher (2018)												
Courtenay (2016)												

Comparison design	Validity of results						Results			Generalisability	
	Q1	Q2	Q3	Q4	Q5	Q6	Q7	Q8	Q9	Q10	Q11
Cosentino et al. (2021)											
Rushton et al. (2021)											
Drenkard (2008)											

Qualitative design	Design and method						Ethics	Validity of results		
	Q1	Q2	Q3	Q4	Q5	Q6	Q7	Q8	Q9	Q10
Kratzke et al. (2023)										
Goodrich (2012)										
Dewar and Cook (2014)										
Knudsen et al. (2023)										
de Senneville et al. (2022)										
Schrøder et al. (2022)										
Vanstone et al. (2020)										
Stacey et al. (2020)										
Nissim et al. (2019)										
Allen et al. (2020)										
Trygg Lycke, Airosa, and Lundh (2023)										
McManus and Robinson (2022)										
Knudsen et al. (2021)										
Curtis et al. (2017)										
Franco et al. (2024)										
Maben et al. (2021)										
Drobinska (2020)										
Orellana‐Rios et al. (2018)										
MacArthur (2014) and MacArthur et al. (2017)										
Sands, Stanley, and Charon (2008)										
Adamson et al. (2018)										
Small, Feldman, and Oldfield (2017)										
Flowers et al. (2018)										
Drenkard (2008)										

*Note*: Colour codes: green= ‘yes’, red= ‘no’ and grey= ‘do not know’.

**TABLE 4:** Identified outcome measures related to organisational compassion targeting HPs.

Outcome measures	Number of studies	Studies
Organisation		
Vacancy	1	Drenkard (2008)
Retention	1	Dreher (2018)
Turnover		
Nursing	4	Flanders et al. (2020), Drenkard (2008), Rushton et al. (2021) and Dreher (2018)
Staff	1	Courtenay (2016)
Organisational commitment	2	Cosentino et al. (2021) and Landry et al. (2018)
Pride in the organisation	1	Drenkard (2008)
Trust in the organisation	1	Landry et al. (2018)
Healthcare environment	1	Drenkard (2008)
Work enforcement	1	Orellana‐Rios et al. (2018)
Workplace culture		
Culture of kindness	1	Landry et al. (2018)
Culture of compassion	2	Schrøder et al. (2022) and Knudsen et al. (2023)
Professional culture	6	Warriner, Hunter, and Dymond (2016), Drobinska (2020), Henderson et al. (2022), Kratzke et al. (2023), Nissim et al. (2019) and Allen et al. (2020)
Management		
Openness to discuss and address work issues	1	Stacey et al. (2020)
Support		
Allocation of protected time for self‐care	2	Burnet et al. (2023) and Kratzke et al. (2023)
Acknowledgment of employee well‐being	6	de Senneville et al. (2022), Nissim et al. (2019), Burnet et al. (2023), Goodrich (2012), Drobinska (2020) and Kratzke et al. (2023)
Job/work		
Satisfaction	4	Copeland (2021), Drenkard (2008), Vanstone et al. (2020) and Orellana‐Rios et al. (2018)
Productivity	1	Franco et al. (2024)
Employee engagement	2	Flanders et al. (2020) and Courtenay (2016)
Work engagement	1	Rushton et al. (2021)
Work enjoyment	1	Orellana‐Rios et al. (2018)
Work life	1	Warriner, Hunter, and Dymond (2016)
Promotion opportunities	1	Drenkard (2008)
Workload	1	Drenkard (2008)
Breaks during work shifts	1	Maben et al. (2021), Orellana‐Rios et al. (2018) and Franco et al. (2024)
Involvement in decision‐making	1	Dewar and Cook (2014)
Team/colleagues/human‐to‐human		
Relationships with co‐workers	10	Nissim et al. (2019), Small, Feldman, and Oldfield (2017), Allen et al. (2020), Drenkard (2008), Duncan et al. (2011), MacArthur (2014), MacArthur et al. (2017), McManus and Robinson (2022), Orellana‐Rios et al. (2018), Knudsen et al. (2023), Franco et al. (2024)
Peer support	5	Drenkard (2008), Stacey et al. (2020), Curtis et al. (2017), Dewar and Cook (2014) and Schrøder et al. (2022)
Co‐worker support	1	Schorch et al. (2021)
Co‐worker sensitivity, recognition and awareness	10	Knudsen et al. (2021), Dewar and Cook (2014), Vanstone et al. (2020), Shah, Lambrecht, and O'Callaghan (2017), Schrøder et al. (2022), Trygg Lycke, Airosa, and Lundh (2023), Knudsen et al. (2023), Franco et al. (2024), Schorch et al. (2021) and Maben et al. (2021)
Team collaboration	2	Goodrich (2012) and Shah, Lambrecht, and O'Callaghan (2017)
Team roles	1	Small, Feldman, and Oldfield (2017)
Team morale	2	Flowers et al. (2018) and Schorch et al. (2021)
Team values	2	MacArthur (2014), MacArthur et al. (2017) and Flowers et al. (2018)
Team communication	4	Copeland (2021), Dewar and Cook (2014), Orellana‐Rios et al. (2018) and Knudsen et al. (2023)
Critic dialogue	1	Curtis et al. (2017)
Discussion of work issues	2	Dewar and Cook (2014), MacArthur (2014) and MacArthur et al. (2017)
Professional reflections	2	Adamson et al. (2018) and Burnet et al. (2023)
Conflict management	2	Nissim et al. (2019) and Orellana‐Rios et al. (2018)
Openness and showing vulnerability (attitude)	4	Shah, Lambrecht, and O'Callaghan (2017), Dewar and Cook (2014), Adamson et al. (2018) and Maben et al. (2021)
Acknowledgement (attitude)	1	Flowers et al. (2018)
Hierarchy	3	Small, Feldman, and Oldfield (2017), Goodrich (2012) and Maben et al. (2021)
Interpersonal trust	3	Dewar and Cook (2014), Maben et al. (2021), MacArthur (2014) and MacArthur et al. (2017)
Interpersonal connection	6	Knudsen et al. (2021), Vanstone et al. (2020), McManus and Robinson (2022), Orellana‐Rios et al. (2018), Small, Feldman, and Oldfield (2017), Sands, Stanley, and Charon (2008)
Human presence	2	Drenkard (2008) and Maben et al. (2021)
Sense of shared humanity	3	Stacey et al. (2020), Drobinska (2020) and Franco et al. (2024)
Sense of community	4	Adamson et al. (2018), Stacey et al. (2020), Maben et al. (2021) and Franco et al. (2024)
Acts of compassion (compassionate behaviour)	12	Landry et al. (2018), Vanstone et al. (2020), Stacey et al. (2020), Curtis et al. (2017), Goodrich (2012), Allen et al. (2020), Dewar and Cook (2014), Maben et al. (2021), Trygg Lycke, Airosa, and Lundh (2023), Knudsen et al. (2023), Franco et al. (2024) and Adamson et al. (2018)

### 2.7 Appraisal

Inspired by Cochrane's Handbook for Systematic Reviews of Interventions (Chandler et al. 2019), we developed a data extraction template (Supporting Information 2). The template was tested on a few studies by C.L.N. and L.N.‐K. before application. In an independent dual process, C.L.N. extracted data from all included studies, while L.N.‐K. and C.T. shared the full workload. Inconsistencies were discussed between the authors until agreement was reached. Further, the authors independently assessed the quality of included studies. This assessment was used only as a marker for study quality in the review as our goal was to present the current state of knowledge (Campbell et al. 2020; Page et al. 2021). We used the critical appraisal skills programme (CASP) tool, which consists of 10–12 assessment questions, depending on the study design, to systematically evaluate the trustworthiness, results and overall value and relevance of each study (Long, French, and Brooks 2020). To illustrate this, each question was assigned a colour code: green for ‘yes’, red for ‘no’ and grey for ‘do not know’. Inconsistencies in this regard were discussed until agreement was reached between the two authors assessing the particular study. Only parts of the studies addressing organisational outcomes relevant to this review were assessed and included. This means that if studies, for instance, used a mixed methods design, but only reported on organisational outcomes in the qualitative part, the study quality was only assessed according to the CASP for qualitative studies.

The included studies varied in quality, as shown in Table 3. Most qualitative studies faced challenges related to ethics, researcher–participant relationships, and sometimes lacked rigour in data analysis and presentation. Many quantitative studies had significant methodological challenges, including failure to report statistical significance, small sample sizes, lack of adjustment for potential confounders and use of nonvalidated questionnaires. Additionally, the complexity of multifaceted interventions made proper design, testing and evaluation difficult. These limitations will be reported alongside the review findings (Campbell et al. 2020).

### 2.8 Data Synthesis

Due to the heterogeneity and risk of biases in the included studies, we decided not to conduct a meta‐analysis. Instead, we performed a content analysis, which entails systematic reading and coding of a body of text (among other sources), including both qualitative and quantitative content (Krippendorff 2018). Adopting an inductive content analysis method inspired by Graneheim and Lundman (2004), C.L.N. and C.T. carried out the analysis through ongoing discussion. Initially, the content areas were determined based on the similarities between the study interventions (Campbell et al. 2020). The meaning units within each area were coded and grouped into categories (not necessarily exclusive) at a low interpretation level (Campbell et al. 2020; Graneheim and Lundman 2004). Subsequently, we elevated the level of interpretation slightly, connecting the underlying meanings within categories to create an overarching descriptive theme (Graneheim, Lindgren, and Lundman 2017).

## 3 Findings

Based on the content analysis of included studies, three themes with categories (shown in parentheses) emerged:

1. Cultivating a caring mind and culture, through mindfulness, resilience and compassion training (*Cultivating the self is cultivating others; Workflow‐integrated practices that intend to foster a more nurturing environment and relationships*; and *The fundamental role of management in changing cultures*)
2. Fostering collective noticing, feeling and responding, through sharing rounds and narrative writing (*Creating a psychologically safe space for sharing that connects humans*; and *Promoting team building through compassionate listening and acknowledgment*)
3. Building community and a nurturing environment, through education, leadership and human care (*Fostering well‐being around elements that interface with workflows*; and *Promoting a sense of community in the workplace*).

### 3.1 Cultivating a Caring Mind and Culture, Through Mindfulness, Resilience and Compassion Training

Fifteen studies investigated the impact of (elements of) cultivating mindfulness, resilience, and/or (self‐)compassion. Among these, six were designed and facilitated as 6–10 weekly, in‐person group sessions, mostly including meditation homework (Drobinska 2020; Knudsen et al. 2021, 2023; Nissim et al. 2019; Orellana‐Rios et al. 2018; Warriner, Hunter, and Dymond 2016). The remaining nine studies include supervision for novice nurses (Stacey et al. 2020), workflow‐integrated practices (Copeland 2021; Franco et al. 2024), workshops supporting digital tools (Curtis et al. 2017; Trygg Lycke, Airosa, and Lundh 2023; Rushton et al. 2021), yearly courses (Kratzke et al. 2023) and drop‐in at a wellness clinic (Duncan et al. 2011) and morning conferences (Burnet et al. 2023). Most intervention evaluations include qualitative investigations, typically marked by rigorous methodologies (see Table 3). Any limitations encountered are detailed below.

#### 3.1.1 Cultivating the Self is Cultivating Others

Overall, qualitative evaluations of mindfulness, resilience and self‐compassion training reveal that HPs, through enhanced self‐awareness and moment‐to‐moment presence, increased their ability to focus on their colleagues (and patients). This cultivated compassionate behaviours that fostered a sense of shared humanity (Drobinska 2020), improved interpersonal understanding and communication (Knudsen et al. 2021, 2023; Trygg Lycke, Airosa, and Lundh 2023) and better relationships (Knudsen et al. 2023; Nissim et al. 2019), including heightened empathy and compassion during challenging interactions (Nissim et al. 2019). Compassionate behaviour was characterised by more frequent greetings and increased eye contact with colleagues (and patients; Trygg Lycke, Airosa, and Lundh 2023), allowing breaks during work (Knudsen et al. 2023; Orellana‐Rios et al. 2018) and having greater awareness of avoiding transmitting and absorbing stressful behaviour (Knudsen et al. 2023). These group interventions positively influenced the atmosphere among participants, fostering a sense of security, shared understanding and connection over time (Knudsen et al. 2023; Nissim et al. 2019; Trygg Lycke, Airosa, and Lundh 2023). However, establishing a shared understanding at the team level seemed more challenging because only ‘a smaller part’ of the employees participated in the intervention (Knudsen et al. 2023).

Moreover, mindfulness, resilience and (self‐)compassion interventions resulted in improved feelings of belonging for novice nurses who received compassion‐based supervision (Stacey et al. 2020) and better team communication (Curtis et al. 2017; Orellana‐Rios et al. 2018) and conflict management (Orellana‐Rios et al. 2018) among interdisciplinary HPs who practised self‐compassion. Orellana‐Rios et al. (2018) also found a significant increase in HPs' levels of work enjoyment, from a mean score of 7.75–8.19 (*p* = 0.005) but nonsignificant increases in their levels of work satisfaction (7.75–8.19, *p* = 0.117) and work enforcement (7.83–8.15, *p* = 0.274). Further, a significantly higher level of work engagement (from a mean score of 4.97–5.28, *p* = 0.001) and nonsignificant lower turnover intentions (2.75–2.48, *p* = 0.05) were observed among nurses who participated in the resilience programme by Rushton et al. (2021). Moreover, in their descriptive survey evaluation, Warriner, Hunter, and Dymond (2016) revealed that most HPs used their learned skills daily or weekly at 4–6 months postintervention and that this positively affected their work life (*n* = 21, 91%) and the workplace culture (*n* = 13, 59%). However, prevalent hospital cultures, such as ‘stiff upper lip’ and ‘patient first’ mentalities, where HPs cannot exhibit emotions and prioritise their well‐being, respectively, appear to be challenging factors for implementation (Drobinska 2020; Nissim et al. 2019).

#### 3.1.2 Workflow‐Integrated Practices That Intend to Foster a More Nurturing Environment and Relationships

Among military hospital HPs who regularly received guided meditations in a drop‐in wellness clinic, gradual improvements in the perception of ‘more ease in relationships with co‐workers’ were observed (Duncan et al. 2011). This was based on a self‐made survey and involved no statistical significance reports. Further, the evaluation of drop‐in morning conferences, involving self‐compassion practices, demonstrated that self‐compassion does not align with the prevalent culture of surgical residency and the personalities of surgical residents (Kratzke et al. 2023). However, with time and programme adjustment, surgical residents felt safer disclosing their vulnerability and gained more confidence in sharing during the course (Kratzke et al. 2023).

Conversely, Franco et al.'s (2024) study, focusing on social connections, revealed that fostering social connections through weekly lunch meetings and mindfulness exercises at work led to feelings of shared humanity and community and stronger relationships. Fostering human interconnection facilitated the integration of self‐compassion into practice and created a caring atmosphere (Franco et al. 2024). Such an atmosphere made it easier for individual HPs not only to share emotions and understand colleagues, but also recognise and articulate needs. They were assisting each other in taking breaks during work, while also setting compassionate boundaries (Franco et al. 2024). Copeland (2021) likewise delivered (5 min) mindfulness exercises in the workplace but revealed no improvements in teamwork perception among the participating nurses. The same applied to the groups practising 5‐min outside breaks and 5‐min journaling, but ran contrary to nurses practising 5‐min gratitude (significant improvements for communication scores, from a mean score of 8.5–6.25, *p* = 0.037; Copeland 2021). Moreover, there were no significant differences between the pre–post levels of job satisfaction for any of the groups (Copeland 2021).

#### 3.1.3 The Fundamental Role of Management in Changing Cultures

As previously noted, overcoming a pervasive ‘professional culture’ can present significant challenges. Additionally, the acknowledgment and support from management play a fundamental role in the success of mindfulness, resilience and (self‐)compassion initiatives. Drobinska (2020) observed a high attrition rate (66%), primarily attributable to the absence of management support, that made HPs feel undervalued in relation to their contributions. Conversely, participants in Burnet et al.'s (2023) and Kratzke et al.'s (2023) studies among surgical residents perceived the allocation of protected time for participation to be valuable for sharing experiences and reflections. Protected time indicated that their managers prioritised employee well‐being, which proved essential in increasing appreciation for the self‐compassion initiatives. This management support, along with course adjustments, also fostered the sense of feeling more secure and greater confidence among surgical residents to share thoughts and experiences during the courses (Kratzke et al. 2023). Additionally, participants in Nissim et al.'s (2019) study noted an improved acknowledgment from management through the intervention. This symbolised a commitment to the shared responsibility for employee well‐being within the organisation.

### 3.2 Fostering Collective Noticing, Feeling and Responding, Through Sharing Rounds and Narrative Writing

Ten studies evaluated group sharing, based on real‐life cases and narrative writings. Among these, six reported on compassion‐based rounds (Flowers et al. 2018; McManus and Robinson 2022) and Schwartz Rounds, primarily in the United Kingdom (Allen et al. 2020; Goodrich 2012; Maben et al. 2021; Shah, Lambrecht, and O'Callaghan 2017). The ‘Schwartz Round’ is a concept originally developed by the lawyer, Kenneth Schwartz, who died from lung cancer, and aims to support and promote the well‐being of HPs. Schwartz desired to create a space where HPs could meet and discuss the emotional and social aspects of their work (Shah, Lambrecht, and O'Callaghan 2017). The features of Schwartz Rounds are the personal, work‐related cases presented by a couple of HPs, discussed with a panel of HPs, and finally opened up for the observing audience of HPs to share their thoughts and topic‐related experiences (Goodrich 2012). Moreover, four studies, primarily within paediatric settings, experimented with Narrative Medicine/writing that involved writing exercises that were either quiet (Sands, Stanley, and Charon 2008), excessive (Adamson et al. 2018; Cosentino et al. 2021) or based on a prompt (Small, Feldman, and Oldfield 2017). These practices typically included shared reflections.

The evaluations of the sharing and writing interventions mostly involved thorough qualitative investigations (see Table 3). Additionally, Cosentino et al. (2021) employed a comparison design, utilising multiple validated questionnaires and conducted robust pre–post intervention group and control group calculations. Shah, Lambrecht, and O'Callaghan (2017), on the contrary, conducted a more descriptive, formative evaluation of their Schwartz Round intervention. They used unspecified questionnaires and presented percentages of agreement and free‐text comments, but did not perform statistical significance calculations.

#### 3.2.1 Creating a Psychologically Safe Space for Sharing That Connects Humans

Overall, the sharing round evaluations showed that getting together to share and discuss experiences provided a psychologically safe space among HPs for mirroring and sharing emotions (Allen et al. 2020; Goodrich 2012; Maben et al. 2021; Shah, Lambrecht, and O'Callaghan 2017). This practice resulted in improved intercolleague openness, understanding and connection (Goodrich 2012; Maben et al. 2021; Shah, Lambrecht, and O'Callaghan 2017) and increased tolerance, trust and communication (Maben et al. 2021). Further, it fostered empathy (Allen et al. 2020; Goodrich 2012; Maben et al. 2021) and kind and compassionate behaviours (Maben et al. 2021). Moreover, HPs who practised narrative writing experienced getting to know their colleagues even better, both professionally and personally (Adamson et al. 2018; Sands, Stanley, and Charon 2008; Small, Feldman, and Oldfield 2017), resulting in greater human relating and sharing of perspectives (Sands, Stanley, and Charon 2008), vulnerability and work‐related issues (Adamson et al. 2018). Getting to know each other fostered a greater sense of community and shared humanity (Adamson et al. 2018; Maben et al. 2021; Small, Feldman, and Oldfield 2017) and flattened hierarchy (Goodrich 2012; Small, Feldman, and Oldfield 2017). Further, the level of organisational (continuance) commitment increased significantly among HPs in the excessive writing group (*Z* = −3.357, *p* = 0.001), compared to the control group, in the study by Cosentino et al. (2021). However, it was also demonstrated that Schwartz Rounds could be (in place for) too short a time and the participant group could be too broad for proper and confident disclosure of emotions (Allen et al. 2020). This also reflected that some of the HPs perceived an expectation to suppress their emotions while they were working (Allen et al. 2020).

#### 3.2.2 Promoting Team Building Through Compassionate Listening and Acknowledgment

The study by Flowers et al. (2018) shows that HPs appreciated engaging in discussions on team values and care during compassion‐based rounds of taking and giving care. In overall statements, they reported feeling more valued as integral members of the team when being complimented during the intervention. This positively affected the team morale (Flowers et al. 2018). Further, HPs, in McManus and Robinson's (2022) study, appreciated addressing their patients' and relatives' emotional, psychological and spiritual needs during rounds with patients, relatives and colleagues. They expressed an enhanced feeling of being heard and an increased sense of connection as a team. This resulted in improved relationships with patients, relatives and colleagues (McManus and Robinson 2022).

### 3.3 Building Community and a Nurturing Environment, Through Education, Leadership and Human Care

Thirteen studies revolved around workplace initiatives to foster human care, teamwork and community. These included interventions that targeted elements which interfaced with workflows and involved kind communication (de Senneville et al. 2022; Landry et al. 2018) and human caring in practice (Drenkard 2008; MacArthur 2014; MacArthur et al. 2017; Vanstone et al. 2020) and after adverse events (Schrøder et al. 2022). Further, initiatives included both educational (Courtenay 2016; Dreher 2018) and leadership (Dewar and Cook 2014) programmes and joint social activities, allowing people to get to know and communicate with one another (Flanders et al. 2020; Henderson et al. 2022; Schorch et al. 2021). Among these studies, an almost equal number employed quantitative and qualitative approaches to evaluate their interventions. Thus, about half of the evaluations were based on survey data, with approximately half of those studies not providing reports of statistical significance (presented in detail below).

#### 3.3.1 Fostering Well‐Being Around Elements That Interface With Workflows

Team‐based interventions aimed at fostering integrated care involve accommodating dying patients' and relatives' wishes (Vanstone et al. 2020) and lowering the workload and increasing human care in the workplace (Drenkard 2008). Further, these interventions involve the promotion of peer (Schrøder et al. 2022) and nursing leader (Dewar and Cook 2014) support systems and kindness (de Senneville et al. 2022), and fostering clinical leadership and shared values among HPs (MacArthur 2014; MacArthur et al. 2017). de Senneville et al. (2022) implemented a communication tool called KISBAR (Kindness, Introduction, Situation, Background, Assessment and Recommendation). Facilitated by an emotional support person, KISBAR was applied to promote kind communication during labour ward handovers. This new procedure resulted in feelings of increased organisational acknowledgment of junior doctors' well‐being and safety in communication, which was in contrast to previous experiences (de Senneville et al. 2022). These overall statements, however, were not further elaborated on in the analysis. Similarly, MacArthur (2014) and MacArthur et al. (2017) found communication improvements among HPs who participated in their Compassionate Care programme concerning human beliefs, values and the meaning of compassionate care. They reported enhanced communication, along with more confidence in opening work‐related discussions with colleagues (MacArthur 2014; MacArthur et al. 2017).

Moreover, lowered workload and increased human care led to significant improvements (*p* = ≤ 0.05) in nurses' perceptions of the healthcare environment (change score = 0.09), which included workload (0.21) and relationships with co‐workers (0.21; Drenkard 2008). The nurses felt able to care for each other, to a greater extent, and experienced support from peers and leaders to spend more time with patients, which fostered job satisfaction. The other improvements observed, regarding relationships with physicians, pride in the organisation, promotional opportunities and relationships with other nurses, were not statistically significant (Drenkard 2008). Similarly, in the study by Vanstone et al. (2020), HPs reported strengthened colleague connections, along with enhanced meaning in clinical work, by accommodating dying patients' and their relatives' wishes. Furthermore, increased attention towards colleagues' suffering, the fostering of greater sharing and trust (Dewar and Cook 2014; Schrøder et al. 2022), and a culture of compassion (Schrøder et al. 2022), security and continuity (Dewar and Cook 2014) were observed after enhanced peer and nursing leader support. Additionally, nursing leader support resulted in increased confidence and communication skills, leading to reduced judgmental attitudes and improved relationships (Dewar and Cook 2014). Challenges, however, include integrating peer support initiatives into existing natural peer structures, involuntary participation (Schrøder et al. 2022) and nurses' feelings of low achievement and influence in their work (Dewar and Cook 2014).

#### 3.3.2 Promoting a Sense of Community in the Workplace

The implementation of initiatives to enhance social and emotional well‐being, through relational skills and social and creative activities, mostly resulted in positive yet often nonsignificant outcomes for HPs. Utilising a self‐made questionnaire for evaluating their wellness programme on education, social support and self‐care, Schorch et al. (2021) observed increases in co‐worker support, recognition, teamwork morale and job satisfaction among care assistants. However, only teamwork morale was statistically significant (improved from medium (3) to high (4) on a 5‐point Likert scale, SD = 0.50; Schorch et al. 2021). Moreover, a nonsignificant increase in employee engagement, from a mean score of 4.15–4.18 (*p* = 0.67), and a nonsignificant decrease in nursing turnover scores by 6% (*p* = 0.22) were reported by Flanders et al. (2020). They evaluated an art, music and pet therapy intervention among paediatric intensive care nurses. Additionally, Landry et al. (2018) observed improvements in HP's perception of the culture of kindness, work commitment and workplace trust in their study testing campaigns for promoting kindness. They linked the improved ‘culture of kindness’ to the fact that HPs smiled, had eye contact and greeted each other to a greater extent (Landry et al. 2018). However, no statistical significance calculations were performed on these results, and it remains unclear what questionnaire(s) they used for evaluation.

Henderson et al. (2022), in contrast, found no significant changes in the overall perception of workplace culture, which was rated as continuously satisfied, during the first 3‐year period (2015–2017) of their intervention. Their intervention encompassed ‘Compassion Cafés’, internal and external peer support, open forums and feedback for nurses that aimed to improve their clinical capital (emotional, social and clinical competencies). However, a statistically significant decrease in the overall perception of workplace culture in 2019 was observed (Henderson et al. 2022), but was not further elaborated on in the text. Moreover, Dreher (2018) found increases in retention rates by 40%–44% per month over 3 months, compared to the same months in the preceding year, when evaluating an educational self‐care programme among certified nursing assistants at a veteran's nursing home. This contrasts with Courtenay (2016), who reported overall turnover increases from 19.6% to 21.3% for all employees, 13.9% to 15.7% for full‐time and part‐time employees and 15.7% to 19.5% for full‐time and part‐time nurses (Table 2), evaluating education, relaxation and role‐play workshops. Finally, Courtenay (2016) reported an overall increase in employee engagement, from 51st to 78th percentile ranking in Gallup's Hospital Level Database. Neither Dreher (2018) nor Courtenay (2016) reported on statistical significance in their studies.

## 4 Discussion

This systematic review provides valuable insights into the existing body of knowledge and research practices regarding organisational compassion to enhance HPs' well‐being. A range of interventions and outcomes were found (see Tables 2 and 4). However, as hypothesised, compassion‐based interventions, targeted at the organisational level, are quite new, representing a burgeoning initiative, particularly in healthcare organisations in the United Kingdom and the United States. Consequently, it is challenging to fully realise at this stage our initial goal to establish a foundational framework for fostering compassion in healthcare organisations. However, we identified a fundamental component—that is, the value of human interconnectedness—which is key to the promotion of organisational compassion that benefits HPs.

Firstly, this review found that it is important that managers show commitment to organisational initiatives that aim to improve employees' well‐being. Their support is essential in catalysing and facilitating organisational changes, including establishing psychological safety, as demonstrated in this review. This aligns with the experiences of nurses and physicians practising in the United States, who prefer that managers prioritise organisational (to individual) solutions (Aiken et al. 2023). In the United Kingdom, ‘The Schwartz Centre Healing Healthcare Initiative’ has been developed to guide managers in nurturing HPs' well‐being (Lown et al. 2024). The framework's principles broadly align with our findings, and include psychological safety to create a safe, trusted environment; involvement of HPs in decision‐making; team cohesion and collaboration and, interpersonal trust (Lown et al. 2024). Nurturing HP's well‐being entails support for both their personal and professional development (Beardsmore and McSherry 2017) and the provision of opportunities to reflect on the emotional and social aspects of clinical care (Seager 2014). Indeed, taking breaks to process such stimuli and to ‘check‐in’ with oneself and each other are essential, to maintain a receptive state of mind and sustained well‐being (Seager 2014). Such actions were also practised and valued by HPs in this review, based on their cultivation of mindfulness and self‐compassion (Franco et al. 2024; Knudsen et al. 2023; Orellana‐Rios et al. 2018). For example, taking regular breaks during work resulted in experiences of being more productive (Franco et al. 2024). Additionally, the current review indicates that it is beneficial to allocate protected time for physicians to prioritise their well‐being (Burnet et al. 2023; Kratzke et al. 2023), as well as time and space for value‐related activities in the workplace (Flowers et al. 2018; MacArthur 2014; MacArthur et al. 2017). In fact, working in value‐discrepant environments has been linked with poorer HP health outcomes and can hinder HPs' ability to act compassionately (Pavlova et al. 2023).

Compassionate behaviour seems important in creating nurturing work environments and well‐being. Various studies in this review reveal characteristics of compassionate behaviour. This includes, for instance, smiling, greeting, active listening, communicating kindly, having eye contact, being nonjudgemental and empathic and setting boundaries. Such behaviour was often shown to create a positive atmosphere and improve interpersonal connections and relationships among HPs. Interpersonal connections were primarily fostered through interventions that physically brought HPs together to do joint activities. These overall included social events inside and outside the workplace and activities at the workplace to collectively share work‐related experiences and emotions. Especially group‐based Schwartz Rounds and Narrative Medicine and more individual‐oriented mindfulness and self‐compassion training, were applied to meet and share experiences and practice the mind respectively. Through regular meetings and shared activities, HPs have the opportunity to get to know each other, work together and connect as humans. This seems to foster a sense of community and shared humanity. In particular, the qualitative study findings in this review suggest that improved interpersonal connections that foster a sense of community and shared humanity among HPs are a protective factor for well‐being. In some of these studies, the suggested link between the cultivation of mindfulness and self‐compassion and the promotion of a sense of shared humanity and community can be explained by the nature of contemplative practices. Self‐compassion, including mindfulness, intends to cultivate a mental attitude and motivation to help others (Germer and Neff 2019). This can for instance include loving‐kindness practices, which expand one's focus to wish well to all fellow beings (Feldman and Kuyken 2011). As such, self‐compassion can foster pro‐social motivations that enhance the feelings of human interconnectedness and love, which influence our behaviour (Orellana‐Rios et al. 2018). From this view, human interconnectedness manifests universally and globally before it delves into relatedness and relationships and then into the emotional aspect of being interconnected. This fosters mutual comfort and well‐being (Dong 2020). However, Sinclair et al. (2017), on the other hand, argue that self‐compassion should be viewed as a combination of common aspects of self‐care, healthy self‐attitude and self‐awareness, rather than as a distinct construct in and of itself. They suggest combining self‐attitude and self‐care with compassion can undermine compassion's inherently relational, prosocial, action‐oriented and selfless nature (Sinclair et al. 2017). Furthermore, they highlight a lack of research on how self‐compassion interventions influence those receiving care, despite this being a key outcome of such interventions (Sinclair et al. 2017).

In quantitative investigations, on the contrary, interpersonal connections or relationships seem to be a more or less overlooked factor and may constitute a potential confounding factor in some studies. This may be because of a general lack of understanding of the definition of organisational compassion and thus how interventions that affect individual employees, groups and organisational culture should be measured. Indeed, this challenge has led to the utilisation of multiple measurement tools for evaluation, resulting in increased intervention dropout among participants (Cosentino et al. 2021). Another challenge, and the most significant limitation of the included quantitative studies, is that nearly half did not report on statistical significance. Further challenges of some included studies include measuring or assessing cultural impacts at the time of, or shortly after, intervention completion. This was primarily done based on short intervention periods or interventions conducted in isolation from natural workflows, with limited participation by the entire workforce. Moreover, most studies used noncomparative designs, and several tested multifaceted interventions making it difficult to determine which (if these) component(s) had led to the specific outcome(s) observed. Thus, careful consideration is necessary when determining the timeframe, intervention content, study design, number of participants and suitable measurement tools for evaluation. Addressing the latter may necessitate the development of a compassion measurement tool tailored specifically to organisational contexts, which is also highlighted by Mascaro et al. (2020). Overall, more rigorous, preferably comparative (control/waitlist control), studies on compassion‐based interventions targeting the organisational level are needed.

The idea of social capital in the workplace is, according to Taylor (2013), to build social relationships between colleagues that benefit both the organisation and the individual. From this perspective, trustful relationships foster the interpersonal sharing of information and offer support, through a shared language and shared goals. This promotes resilience and better facilitation of work‐related activities and learning within the organisation (Taylor 2013). Henderson et al. (2022), in this review, seem to expand this concept by adding the aspect of emotional well‐being and using the broader term ‘clinical capital’. However, ‘collegial relationships’ may be a more nuanced phenomenon, given the presence of diverse sub‐groups and cultures among HPs (Hall 2005) and the distinction between peers and ‘other colleagues’ (Franco et al. 2024). For instance, in this review, physicians (obstetricians and surgeons) appear to face challenges of uncertainty and insecurity when it comes to sharing information, thoughts and feelings, particularly in the presence of their superiors or bosses (de Senneville et al. 2022; Kratzke et al. 2023). Literature within this field attests to the fact that traditional medical culture can restrict physicians, especially surgeons, from expressing themselves (Voultsos 2021). Among physicians, studies indicate that surgeons tend to have lower empathy (Pavlova et al. 2022). They often suppress or regulate emotions to operate effectively (Childers and Arnold 2019) or to comply with organisational rules, potentially leading to a perception of invulnerability (Voultsos 2021). This can explain why surgeons, in Kratzke et al.'s (2023) example, experienced discrepancies between self‐compassion, which revolves around vulnerability and suffering, and the perceived culture and personality of surgeons.

To help us understand diversity in human perceptions and actions, we have chosen to draw on the thinking of Heidegger (1962). Central to his work on ‘being‐in‐the‐world’ (‘In der Welt Sein’) are the concepts of ‘situatedness’ and ‘moods’. With these concepts, we can deepen our understanding of how humans inevitably find themselves immersed in situations where certain things hold significance for them based on their specific moods (Aho 2013). Human existence is primarily shaped by concern for the world and a sense of belonging within it. Therefore, moods cannot be regarded as merely internal feelings or states of mind; rather, they are inseparable from the broader contexts of meaning and shared significance in which humans are embedded (Heidegger 1962). As humans, we thus experience varying levels of significance based on our engagement with certain things, and this guides our attention towards what matters to us, for example, in our work life. This influences our perceptions of the world and thus our motivations for compassion (Gilbert and van Gordon 2023) and creates an atmosphere that shapes our experiences and interactions, influencing our relationship with everything around us (Aho 2013). These psychological processes, grounded in our experiences, thoughts and emotions, and which shape our behaviours, require further investigation to enhance our relational understanding (Gilbert and van Gordon 2023; Iles 2014). For instance, exploring human values and cultures within and among HP subgroups would be valuable, to help us understand motivations (for compassion). Such knowledge can help us build tailored and meaningful compassion initiatives in our healthcare organisations.

### 4.1 Strengths and Limitations

There are strengths and limitations to highlight regarding this review. First, there is a lack of consensus in the literature on the definition of compassion, which poses potential challenges in fully capturing the concept in our literature searches. Nevertheless, by including studies that implicitly address compassion, including recurring concepts, based on the search on compassion‐related terms and the screening of a large number of articles, theses and books, we believe we have captured a wide range of interventions relevant to this review. This includes four theses, two of which are unique and would not have been identified through article searches. However, our approach also points to the fact that, although some of the included studies did not intentionally address well‐being on an organisational level, they were assessed based on such premises, which is a limitation of the review.

Furthermore, it is generally recommended not to search for outcomes (Frandsen, Nielsen, and Eriksen 2022). However, as this review includes all interventions to improve HPs' well‐being that was targeted to the organisational level, a very wide definition of the intervention was used, and thus, outcomes can be difficult to exclude from the search strategy (Frandsen, Nielsen, and Eriksen 2022). Finally, the dual assessment method employed throughout the assessment and selection processes should be noted. Given the elusive nature of compassion, the requirement of consistency between two independent researchers might have been particularly important, resulting in increased reliability and thus validity of the review (Chandler et al. 2019).

## 5 Conclusion

Implementing compassion‐based initiatives targeting the organisational level to improve HPs' well‐being is important and represents a burgeoning initiative in healthcare organisations, primarily in the United Kingdom and the United States. Despite some significant methodological challenges in the included studies, this review strongly indicates that interpersonal connections between HPs, at a human level, are associated with increased well‐being in the workplace. Human interconnectedness, achieved through fostering a caring mind and behaviour, may thus be of key importance in promoting compassion in organisations and, potentially, a more shared culture. Interconnectedness may enhance HPs' capability for compassion, which in turn benefits their well‐being and, ultimately, the patients and relatives they care for. However, more studies are necessary to confirm the results of this review and further contribute to the development of an evidence‐based foundational framework for action. For this purpose, further clarification of the organisational cultural aspect of compassion, including subcultures and values among HPs, would be helpful.

## Author Contributions

**Camilla Littau Nielsen:** funding acquisition, conceptualisation, data curation, methodology, formal analysis, investigation, project administration, validation, writing – original draft, visualisation. **Christina Louise Lindhardt:** conceptualisation, data curation, methodology, investigation, validation, writing – original draft. **Lui Näslund‐Koch:** data curation, investigation, validation, writing – original draft, visualisation, conceptualisation. **Tove Faber Frandsen:** resource, validation, writing – original draft, visualisation, methodology, data curation. **Jane Clemensen:** funding acquisition, conceptualisation, project administration, validation, supervision, writing – review and editing. **Connie Timmermann:** supervision, conceptualisation, data curation, validation, writing – review and editing, formal analysis, investigation, methodology.

## Conflicts of Interest

The authors declare no conflicts of interest.

### Peer Review

The peer review history for this article is available at https://www.webofscience.com/api/gateway/wos/peer‐review/10.1111/jan.16484.

## Supporting information


Supporting Information 1.

Supporting Information 2.

## Data Availability

Data available on request from the authors.

## References

[jan16484-bib-0001] Adamson, K. , S. Sengsavang , A. Charise , S. Wall , L. Kinross , and M. Balkaran . 2018. “Narrative Training as a Method to Promote Nursing Empathy Within a Pediatric Rehabilitation Setting.” Journal of Pediatric Nursing 42: e2–e9. 30007769 10.1016/j.pedn.2018.06.011

[jan16484-bib-0002] Aho, K. A. 2013. “Depression and Embodiment: Phenomenological Reflections on Motility, Affectivity, and Transcendence.” Medicine, Health Care and Philosophy 16: 751–759. 23378190 10.1007/s11019-013-9470-8

[jan16484-bib-0003] Aiken, L. H. , K. B. Lasater , D. M. Sloane , et al. 2023. “Physician and Nurse Well‐Being and Preferred Interventions to Address Burnout in Hospital Practice: Factors Associated With Turnover, Outcomes, and Patient Safety.” Paper presented at the JAMA Health Forum. 10.1001/jamahealthforum.2023.1809 PMC10329209 37418269

[jan16484-bib-0004] Allen, D. , G. Spencer , K. McEwan , et al. 2020. “The Schwartz Centre Rounds: Supporting Mental Health Workers With the Emotional Impact of Their Work.” International Journal of Mental Health Nursing 29, no. 5: 942–952. 32413204 10.1111/inm.12729

[jan16484-bib-0005] American Psychological Association . 2022. Resilience. Washington, DC: American Psychological Association. https://www.apa.org/topics/resilience.

[jan16484-bib-0006] Bardhan, I. , H. Chen , and E. Karahanna . 2020. “Connecting Systems, Data, and People: A Multidisciplinary Research Roadmap for Chronic Disease Management.” MIS Quarterly 44, no. 1: 185–200.

[jan16484-bib-0007] Beardsmore, E. , and R. McSherry . 2017. “Healthcare Workers' Perceptions of Organisational Culture and the Impact on the Delivery of Compassionate Quality Care.” Journal of Research in Nursing 22, no. 1–2: 42–56.

[jan16484-bib-0008] Burnet, G. , C. Platnick , P. Krishnan , et al. 2023. “Muffins and Meditation: Combatting Burnout in Surgical Residents.” Journal of Surgical Education 80, no. 2: 185–193. 36184410 10.1016/j.jsurg.2022.09.005

[jan16484-bib-0009] Campbell, M. , J. E. McKenzie , A. Sowden , et al. 2020. “Synthesis Without Meta‐Analysis (SWiM) in Systematic Reviews: Reporting Guideline.” BMJ 368: l6890. 31948937 10.1136/bmj.l6890 PMC7190266

[jan16484-bib-0010] Chandler, J. , M. Cumpston , T. Li , M. J. Page , and V. Welch . 2019. Cochrane Handbook for Systematic Reviews of Interventions. Hoboken: Wiley. 10.1002/14651858.ED000142 PMC10284251 31643080

[jan16484-bib-0011] Childers, J. , and B. Arnold . 2019. “The Inner Lives of Doctors: Physician Emotion in the Care of the Seriously Ill.” American Journal of Bioethics 19, no. 12: 29–34. 10.1080/15265161.2019.1674409 31746722

[jan16484-bib-0012] Cohen, C. , S. Pignata , E. Bezak , M. Tie , and J. Childs . 2023. “Workplace Interventions to Improve Well‐Being and Reduce Burnout for Nurses, Physicians and Allied Healthcare Professionals: A Systematic Review.” BMJ Open 13, no. 6: e071203. 10.1136/bmjopen-2022-071203 PMC10314589 37385740

[jan16484-bib-0013] Copeland, D. 2021. “Brief Workplace Interventions Addressing Burnout, Compassion Fatigue, and Teamwork: A Pilot Study.” Western Journal of Nursing Research 43, no. 2: 130–137. 32646295 10.1177/0193945920938048

[jan16484-bib-0014] Cosentino, C. , C. D'apice , M. Del Gaudio , et al. 2021. “Effectiveness of Expressive Writing Protocol in Palliative Care Healthworkers: A Quantitative Study.” Acta Bio Medica: Atenei Parmensis 92, no. S2: e2021010. 10.23750/abm.v92iS2.11468. PMC8138804 33855988

[jan16484-bib-0015] Courtenay, T. M. 2016. “Improving Inpatient Experience Utilizing an Empathy Enhancement Program.” Doctoral diss., University of Hawai'i at Manoa.

[jan16484-bib-0016] Curtis, K. , A. Gallagher , C. Ramage , et al. 2017. “Using Appreciative Inquiry to Develop, Implement and Evaluate a Multi‐Organisation ‘Cultivating compassion’ Programme for Health Professionals and Support Staff.” Journal of Research in Nursing 22, no. 1–2: 150–165.

[jan16484-bib-0017] de Senneville, L. L. , A. Brewin , A. Thomas , and K. Calvert . 2022. “A Qualitative Analysis of Adding Kindness Into the ISBAR Handover Tool.” Australian and New Zealand Journal of Obstetrics and Gynaecology 62, no. 6: 901–905. 36097379 10.1111/ajo.13607

[jan16484-bib-0018] Dewar, B. , and F. Cook . 2014. “Developing Compassion Through a Relationship Centred Appreciative Leadership Programme.” Nurse Education Today 34, no. 9: 1258–1264. 24461906 10.1016/j.nedt.2013.12.012

[jan16484-bib-0019] Dong, T. 2020. Mutual Vulnerability: Deepening Human Interconnectedness in Cross‐Racial Relationships. San Marcos: Texas State University.

[jan16484-bib-0020] Doohan, I. , and B. I. Saveman . 2015. “Need for Compassion in Prehospital and Emergency Care: A Qualitative Study on Bus Crash Survivors' Experiences.” International Emergency Nursing 23, no. 2: 115–119. 25257225 10.1016/j.ienj.2014.08.008

[jan16484-bib-0021] Dreher, M. M. 2018. “The Effect of a Compassion Fatigue Awareness and Self‐Care Skills Educational Program on Retention Among Certified Nursing Assistants Working in a Veterans Nursing Home.” Doctoral diss.

[jan16484-bib-0022] Drenkard, K. N. 2008. “Integrating Human Caring Science Into a Professional Nursing Practice Model.” Critical Care Nursing Clinics of North America 20, no. 4: 403–414. 19007706 10.1016/j.ccell.2008.08.008

[jan16484-bib-0023] Drobinska, K. 2020. Compassion in the NHS: An Exploration of the Experiences of Mental Health Staff. United Kingdom: Bangor University.

[jan16484-bib-0024] Duncan, A. D. , J. M. Liechty , C. Miller , G. Chinoy , and R. Ricciardi . 2011. “Employee Use and Perceived Benefit of a Complementary and Alternative Medicine Wellness Clinic at a Major Military Hospital: Evaluation of a Pilot Program.” Journal of Alternative and Complementary Medicine 17, no. 9: 809–815. 21834662 10.1089/acm.2010.0563

[jan16484-bib-0025] Feldman, C. , and W. Kuyken . 2011. “Compassion in the Landscape of Suffering.” Contemporary Buddhism 12, no. 1: 143–155.

[jan16484-bib-0026] Flanders, S. , D. Hampton , P. Missi , C. Ipsan , and C. Gruebbel . 2020. “Effectiveness of a Staff Resilience Program in a Pediatric Intensive Care Unit.” Journal of Pediatric Nursing 50: 1–4. 31669724 10.1016/j.pedn.2019.10.007

[jan16484-bib-0027] Flowers, S. , C. Bradfield , R. Potter , et al. 2018. “Taking Care, Giving Care' Rounds: An Intervention to Support Compassionate Care Amongst Healthcare Staff.” Paper presented at the Clinical Psychology Forum.

[jan16484-bib-0028] Francis, R. 2013. Report of the Mid Staffordshire NHS Foundation Trust Public Inquiry. London: Stationery Office.

[jan16484-bib-0029] Franco, P. L. , M. C. Knox , L. E. Gulbas , and K. Gregory . 2024. “Learning Self‐Compassion Through Social Connection at Work: The Experiences of Healthcare Professionals in a 6‐Week Intervention.” Qualitative Social Work 23, no. 2: 364–381.

[jan16484-bib-0030] Frandsen, T. F. , M. F. B. Nielsen , and M. B. Eriksen . 2022. “Avoiding Searching for Outcomes Called for Additional Search Strategies: A Study of Cochrane Review Searches.” Journal of Clinical Epidemiology 149: 83–88. 35661816 10.1016/j.jclinepi.2022.05.015

[jan16484-bib-0031] Frandsen, T. F. , M. F. B. Nielsen , C. L. Lindhardt , and M. B. Eriksen . 2020. “Using the Full PICO Model as a Search Tool for Systematic Reviews Resulted in Lower Recall for Some PICO Elements.” Journal of Clinical Epidemiology 127: 69–75. 32679315 10.1016/j.jclinepi.2020.07.005

[jan16484-bib-0032] García‐Campayo, J. , A. Barcelo‐Soler , D. Martínez‐Rubio , et al. 2024. “Exploring the Relationship Between Self‐Compassion and Compassion for Others: The Role of Psychological Distress and Wellbeing.” Assessment 31, no. 5: 1038–1051. 37840255 10.1177/10731911231203966 PMC11134997

[jan16484-bib-0033] Germer, C. , and K. Neff . 2019. “Mindful Self‐Compassion (MSC).” In Handbook of Mindfulness‐Based Programmes, edited by I. Itvzak , 357–367. London: Routledge.

[jan16484-bib-0034] Ghahramani, S. , K. B. Lankarani , M. Yousefi , K. Heydari , S. Shahabi , and S. Azmand . 2021. “A Systematic Review and Meta‐Analysis of Burnout Among Healthcare Workers During COVID‐19.” Frontiers in Psychiatry 12: 758849. 34858231 10.3389/fpsyt.2021.758849 PMC8631719

[jan16484-bib-0035] Gilbert, P. , and W. van Gordon . 2023. “Compassion as a Skill: A Comparison of Contemplative and Evolution‐Based Approaches.” Mindfulness 14, no. 10: 2395–2416.

[jan16484-bib-0036] Goodrich, J. 2012. “Supporting Hospital Staff to Provide Compassionate Care: Do Schwartz Center Rounds Work in English Hospitals?” Journal of the Royal Society of Medicine 105, no. 3: 117–122. 22434811 10.1258/jrsm.2011.110183 PMC3308638

[jan16484-bib-0037] Graneheim, U. H. , B.‐M. Lindgren , and B. Lundman . 2017. “Methodological Challenges in Qualitative Content Analysis: A Discussion Paper.” Nurse Education Today 56: 29–34. 28651100 10.1016/j.nedt.2017.06.002

[jan16484-bib-0038] Graneheim, U. H. , and B. Lundman . 2004. “Qualitative Content Analysis in Nursing Research: Concepts, Procedures and Measures to Achieve Trustworthiness.” Nurse Education Today 24, no. 2: 105–112. 14769454 10.1016/j.nedt.2003.10.001

[jan16484-bib-0039] Hall, P. 2005. “Interprofessional Teamwork: Professional Cultures as Barriers.” Journal of Interprofessional Care 19, no. sup1: 188–196. 16096155 10.1080/13561820500081745

[jan16484-bib-0040] Heidegger, M. 1962. Being and Time (1927), trans. J. Macquarrie and E. Robinson. Vol. 58, 282. New York: Harper Collins.

[jan16484-bib-0041] Henderson, A. , M. Takashima , E. Burmeister , P. Strube , and S. Winch . 2022. “Towards the Idea of ‘Clinical Capital’: A Longitudinal Study Exploring Nurses' Dispositions and Workplace Manifestations in an Australian Intensive Care Unit.” Journal of Advanced Nursing 78, no. 11: 3673–3686. 35478413 10.1111/jan.15264 PMC9790404

[jan16484-bib-0042] Iles, V. 2014. “How Good People Can Offer Bad Care: Understanding the Wider Factors in Society That Encourage Non‐Compassionate Care.” In Providing Compassionate Healthcare, edited by S. Shea , R. Wynyard , and C. Lionis , 209–222. London: Routledge.

[jan16484-bib-0043] Knudsen, R. K. , J. Ammentorp , M. H. Storkholm , S. Skovbjerg , C. G. Tousig , and C. Timmermann . 2023. “The Influence of Mindfulness‐Based Stress Reduction on the Work Life of Healthcare Professionals—A Qualitative Study.” Complementary Therapies in Clinical Practice 53: 101805. 37837781 10.1016/j.ctcp.2023.101805

[jan16484-bib-0044] Knudsen, R. K. , T. Gregersen , J. Ammentorp , C. G. Tousig , and C. Timmermann . 2021. “Healthcare Professionals' Experiences of Using Mindfulness Training in a Cardiology Department—A Qualitative Study.” Scandinavian Journal of Caring Sciences 35, no. 3: 892–900. 32852094 10.1111/scs.12906

[jan16484-bib-0045] Kratzke, I. M. , J. L. Barnhill , K. T. Putnam , et al. 2023. “Self‐Compassion Training to Improve Well‐Being for Surgical Residents.” Explorer 19, no. 1: 78–83. 10.1016/j.explore.2022.04.008 35534424

[jan16484-bib-0046] Krippendorff, K. 2018. Content Analysis: An Introduction to Its Methodology. Thousand Oaks: Sage Publications.

[jan16484-bib-0047] Landry, S. , K. Bisson , C. Cook , and L. Morrison . 2018. “How a Culture of Kindness Can Improve Employee Engagement and Patient Experience—And Five Ways to Get There.” Nursing Leadership (1910‐622X) 31, no. 3: 42–47. 10.12927/cjnl.2018.25678 30653454

[jan16484-bib-0048] Lee, M. , and C. Cha . 2023. “Interventions to Reduce Burnout Among Clinical Nurses: Systematic Review and Meta‐Analysis.” Scientific Reports 13, no. 1: 10971. 37414811 10.1038/s41598-023-38169-8 PMC10325963

[jan16484-bib-0049] Long, H. A. , D. P. French , and J. M. Brooks . 2020. “Optimising the Value of the Critical Appraisal Skills Programme (CASP) Tool for Quality Appraisal in Qualitative Evidence Synthesis.” Research Methods in Medicine & Health Sciences 1, no. 1: 31–42.

[jan16484-bib-0050] Lown, B. A. , J. Collier , C. Manning , and K. Gareis . 2024. “The Healing Healthcare Initiative: Guiding Leaders to Heal a Traumatized Workforce.” Paper presented at the Healthcare Management Forum. 10.1177/08404704231204874 37831518

[jan16484-bib-0051] Maben, J. , C. Taylor , E. Reynolds , I. McCarthy , and M. Leamy . 2021. “Realist Evaluation of Schwartz Rounds® for Enhancing the Delivery of Compassionate Healthcare: Understanding How They Work, for Whom, and in What Contexts.” BMC Health Services Research 21: 1–24. 34275468 10.1186/s12913-021-06483-4 PMC8286624

[jan16484-bib-0052] MacArthur, J. 2014. “Embedding Compassionate Care in Local NHS Practice: A Realistic Evaluation of the Leadership in Compassionate Care Programme.” Doctoral diss.

[jan16484-bib-0053] MacArthur, J. , H. Wilkinson , M. A. Gray , and G. Matthews‐Smith . 2017. “Embedding Compassion‐Ate Care in Local NHS Practice: Developing a Conceptual Model Through Realistic Evaluation.” Journal of Research in Nursing 22, no. 1–2: 130–147.

[jan16484-bib-0054] Mascaro, J. S. , M. P. Florian , M. J. Ash , et al. 2020. “Ways of Knowing Compassion: How Do We Come to Know, Understand, and Measure Compassion When We See It?” Frontiers in Psychology 11: 547241. 33132956 10.3389/fpsyg.2020.547241 PMC7561712

[jan16484-bib-0055] McManus, K. , and P. S. Robinson . 2022. “A Thematic Analysis of the Effects of Compassion Rounds on Clinicians and the Families of NICU Patients.” Journal of Health Care Chaplaincy 28, no. 1: 69–80. 32228290 10.1080/08854726.2020.1745489

[jan16484-bib-0056] Melnick, E. R. , C. A. Sinsky , and T. Shanafelt . 2023. “Funding Research on Health Workforce Well‐Being to Optimize the Work Environment.” JAMA 329, no. 14: 1145–1146. 36821127 10.1001/jama.2023.2073

[jan16484-bib-0057] Nissim, R. , C. Malfitano , M. Coleman , G. Rodin , and M. Elliott . 2019. “A Qualitative Study of a Compassion, Presence, and Resilience Training for Oncology Interprofessional Teams.” Journal of Holistic Nursing 37, no. 1: 30–44. 29598225 10.1177/0898010118765016

[jan16484-bib-0058] Orellana‐Rios, C. L. , L. Radbruch , M. Kern , et al. 2018. “Mindfulness and Compassion‐Oriented Practices at Work Reduce Distress and Enhance Self‐Care of Palliative Care Teams: A Mixed‐Method Evaluation of an “On the Job” Program.” BMC Palliative Care 17: 1–15. 10.1186/s12904-017-0219-7 PMC5501358 28683799

[jan16484-bib-0059] Osareme, J. , M. Muonde , C. P. Maduka , T. O. Olorunsogo , and O. Omotayo . 2024. “Demographic Shifts and Healthcare: A Review of Aging Populations and Systemic Challenges.” International Journal of Science and Research Archive 11, no. 1: 383–395.

[jan16484-bib-0060] Page, M. J. , J. E. McKenzie , P. M. Bossuyt , et al. 2021. “The PRISMA 2020 Statement: An Updated Guideline for Reporting Systematic Reviews.” BMJ 372: n71. 33782057 10.1136/bmj.n71 PMC8005924

[jan16484-bib-0061] Page, M. J. , L. Shamseer , and A. C. Tricco . 2018. “Registration of Systematic Reviews in PROSPERO: 30,000 Records and Counting.” Systematic Reviews 7: 1–9. 29463298 10.1186/s13643-018-0699-4 PMC5819709

[jan16484-bib-0062] Pavlova, A. , S. J. Paine , S. Sinclair , A. O'Callaghan , and N. S. Consedine . 2023. “Working in Value‐Discrepant Environments Inhibits Clinicians' Ability to Provide Compassion and Reduces Well‐Being: A Cross‐Sectional Study.” Journal of Internal Medicine 293: 704–723. 36843313 10.1111/joim.13615

[jan16484-bib-0063] Pavlova, A. , C. X. Wang , A. L. Boggiss , A. O'Callaghan , and N. S. Consedine . 2022. “Predictors of Physician Compassion, Empathy, and Related Constructs: A Systematic Review.” Journal of General Internal Medicine 37: 1–12. 10.1007/s11606-021-07055-2 PMC8452146 34545471

[jan16484-bib-0064] Perez‐Bret, E. , R. Altisent , and J. Rocafort . 2016. “Definition of Compassion in Healthcare: A Systematic Literature Review.” International Journal of Palliative Nursing 22, no. 12: 599–606. 27992278 10.12968/ijpn.2016.22.12.599

[jan16484-bib-0065] Rushton, C. H. , S. M. Swoboda , N. Reller , et al. 2021. “Mindful Ethical Practice and Resilience Academy: Equipping Nurses to Address Ethical Challenges, Challenges.” American Journal of Critical Care 30, no. 1: e1–e11. 33385208 10.4037/ajcc2021359

[jan16484-bib-0066] Sands, S. A. , P. Stanley , and R. Charon . 2008. “Pediatric Narrative Oncology: Interprofessional Training to Promote Empathy, Build Teams, and Prevent Burnout.” Journal of Supportive Oncology 6, no. 7: 307–312. 18847073

[jan16484-bib-0067] Schorch, K. , R. Stamm , D. Priddy , and C. Taylor . 2021. “A Wellness Program to Decrease Pediatric Postanesthesia Care Unit Staff Compassion Fatigue.” Journal of Pediatric Health Care 35, no. 5: 526–541. 34112529 10.1016/j.pedhc.2021.04.003

[jan16484-bib-0068] Schrøder, K. , T. Bovil , J. S. Jørgensen , and C. Abrahamsen . 2022. “Evaluation of ‘the Buddy Study’, a Peer Support Program for Second Victims in Healthcare: A Survey in Two Danish Hospital Departments.” BMC Health Services Research 22, no. 1: 1–10. 35477365 10.1186/s12913-022-07973-9 PMC9043887

[jan16484-bib-0069] Seager, M. 2014. “Who Cares for the Carers? Keeping Compassion Alive in Care Systems, Cultures and Environments.” In Providing Compassionate Healthcare: Challenges in Policy and Practice, edited by S. Shea , R. Wynyard , and C. Lionis , 40–53. London: Routledge.

[jan16484-bib-0070] Shah, S. , I. Lambrecht , and A. O'Callaghan . 2017. “Reigniting Compassion in Healthcare: Manaakitia Reflective Rounds.” Internal Medicine Journal 47, no. 6: 674–679. 28266121 10.1111/imj.13420

[jan16484-bib-0071] Sinclair, S. , J. Kondejewski , S. Raffin‐Bouchal , K. M. King‐Shier , and P. Singh . 2017. “Can Self‐Compassion Promote Healthcare Provider Well‐Being and Compassionate Care to Others? Results of a Systematic Review.” Applied Psychology. Health and Well‐Being 9, no. 2: 168–206. 28393485 10.1111/aphw.12086

[jan16484-bib-0072] Small, L. C. , L. S. Feldman , and B. J. Oldfield . 2017. “Using Narrative Medicine to Build Community Across the Health Professions and Foster Self‐Care.” Journal of Radiology Nursing 36, no. 4: 224–227.

[jan16484-bib-0073] Stacey, G. , G. Cook , A. Aubeeluck , et al. 2020. “The Implementation of Resilience Based Clinical Supervision to Support Transition to Practice in Newly Qualified Healthcare Professionals.” Nurse Education Today 94: 104564. 32947209 10.1016/j.nedt.2020.104564

[jan16484-bib-0074] Taylor, R. 2013. “Networking in Primary Health Care: How Connections Can Increase Social Capital.” Primary Health Care 23, no. 10: 34–40.

[jan16484-bib-0075] Trygg Lycke, S. , F. Airosa , and L. Lundh . 2023. “Emergency Department Nurses' Experiences of a Mindfulness Training Intervention: A Phenomenological Exploration.” Journal of Holistic Nursing 41, no. 2: 170–184. 35574608 10.1177/08980101221100091 PMC10230593

[jan16484-bib-0076] Trzeciak, S. , A. Mazzarelli , and C. Booker . 2019. Compassionomics: The Revolutionary Scientific Evidence That Caring Makes a Difference. Pensacola, FL: Studer Group.

[jan16484-bib-0077] Vanstone, M. , M. Sadik , O. Smith , et al. 2020. “Building Organizational Compassion Among Teams Delivering End‐of‐Life Care in the Intensive Care Unit: The 3 Wishes Project.” Palliative Medicine 34, no. 9: 1263–1273. 32519615 10.1177/0269216320929538

[jan16484-bib-0078] Voultsos, P. 2021. “Emotions as Parts of the Inner Lives of Physicians in the Modern Clinical Context.” Aristotle Biomedical Journal 3, no. 1: 16–28.

[jan16484-bib-0079] Warriner, S. , L. Hunter , and M. Dymond . 2016. “Mindfulness in Maternity: Evaluation of a Course for Midwives.” British Journal of Midwifery 24, no. 3: 188–195.

[jan16484-bib-0080] Zhang, Y. Y. , W. L. Han , W. Qin , et al. 2018. “Extent of Compassion Satisfaction, Compassion Fatigue and Burnout in Nursing: A Meta‐Analysis.” Journal of Nursing Management 26, no. 7: 810–819. 30129106 10.1111/jonm.12589

