# Supplementary material for: What is the State of Organisational Compassion‐Based Interventions Targeting to Improve Health Professionals' Well‐Being? Results of a Systematic Review
Source: J Adv Nurs. 2024 Oct 7;81(5):2246–76. doi: 10.1111/jan.16484 (PMC11967289; doi:10.1111/jan.16484)
Supplement: Supplementary file 1 — Supporting Information 1. [file JAN-81-2246-s002.docx]

| **Bibliographic database**  **Supplementary File 1 – Searches** | **Search** | **Results (search 1; 20 Sep. 2022)** | **Results (search 2; 26 Dec. 2023 – time: year 2022 to present)** |
| --- | --- | --- | --- |
| Scopus | **S1)** TITLE-ABS-KEY ( ( ( compassion* OR kindness OR empath* OR sympath* OR genero ) W/3 ( unit* OR principle* OR department* OR organisation* OR communit* OR practice* OR workplace* OR culture* OR environment* OR facilit* OR philosoph* OR organization* OR climate* ) ) AND ( hospi* OR health* ) )  **S2)** TITLE-ABS-KEY ( ( compassion* OR kindness OR empath* OR sympath* OR genero* ) W/3 ( hospi* OR health* ) )  **S3)** ( TITLE-ABS-KEY ( ( compassion* OR kindness OR empath* OR sympath* OR genero* ) W/3 ( hospi* OR health* ) ) ) OR ( TITLE-ABS-KEY ( ( ( compassion* OR kindness OR empath* OR sympath* OR genero ) W/3 ( unit* OR principle* OR department* OR organisation* OR communit* OR practice* OR workplace* OR culture* OR environment* OR facilit* OR philosoph* OR organization* OR climate* ) ) AND ( hospi* OR health* ) ) ) | 1855  3073  **4613** | 547  801  **1253** |
| CINAHL | **S1)** ( Empath* N3 (unit* or department* or organisation* or communit* or practice* or design* or ward* or workplace* or culture* or core* or value* or environment* or institut* or philosop* or principle* or organization* or climate*) ) AND ( health* or hospi* )  **S2)** Empath* N3 (health* or hospi*)  **S3)** ( ( Empath* N3 (unit* or department* or organisation* or communit* or practice* or design* or ward* or workplace* or culture* or core* or value* or environment* or institut* or philosop* or principle* or organization* or climate*) ) AND ( health* or hospi* ) ) OR ( Empath* N3 (health* or hospi*) )  **S4)** ( Sympath* N3 (unit* or department* or organisation* or communit* or practice* or design* or ward* or workplace* or culture* or core* or value* or environment* or institut* or philosop* or principle* or organization* or climate*) ) AND ( health* or hospi* )  **S5)** Sympath* N3 (health* or hospi*)  **S6)** ( (Sympath* N3 (unit* or department* or organisation* or communit* or practice* or design* or ward* or workplace* or culture* or core* or value* or environment* or institut* or philosop* or principle* or organization* or climate*)) AND ( health* or hospi* ) ) OR ( Sympath* N3 (health* or hospi*) )  **S7)** ( Compassion* N3 (unit* or department* or organisation* or communit* or practice* or design* or ward* or workplace* or culture* or core* or value* or environment* or institut* or philosop* or principle* or organization* or climate*) ) AND ( health* or hospi* )  **S8)** Compassion* N3 (health* or hospi*)  **S9)** ( ( Compassion* N3 (unit* or department* or organisation* or communit* or practice* or design* or ward* or workplace* or culture* or core* or value* or environment* or institut* or philosop* or principle* or organization* or climate*) ) AND ( health* or hospi* ) ) OR ( Compassion* N3 (health* or hospi*) )  **S10)** ( Kindness N3 (unit* or department* or organisation* or communit* or practice* or design* or ward* or workplace* or culture* or core* or value* or environment* or institut* or philosop* or principle* or organization* or climate*) ) AND ( health* or hospi* )  **S11)** Kindness N3 (health* or hospi*)  **S12)** ( ( Kindness N3 (unit* or department* or organisation* or communit* or practice* or design* or ward* or workplace* or culture* or core* or value* or environment* or institut* or philosop* or principle* or organization* or climate*) ) AND ( health* or hospi* ) ) OR ( Kindness N3 (health* or hospi*) )  **S13)** ( Genero* N3 (unit* or department* or organisation* or communit* or practice* or design* or ward* or workplace* or culture* or core* or value* or environment* or institut* or philosop* or principle* or organization* or climate*) ) AND ( health* or hospi* )  **S14)** Genero* N3 (health* or hospi*)  **S15)** ( ( Genero* N3 (unit* or department* or organisation* or communit* or practice* or design* or ward* or workplace* or culture* or core* or value* or environment* or institut* or philosop* or principle* or organization* or climate*) ) AND ( health* or hospi* ) ) OR ( Genero* N3 (health* or hospi*) )  **S16)** ((MH "Compassion") OR (MH "Empathy")) AND (MH "Health Facilities+")  **S17)** S3 OR S6 OR S9 OR S12 OR S15 OR S16  Limitation: Language: Norwegian, Danish, Swedish, and English | 394  538  867  67  100  167  715  722  1295  59  45  96  41  90  124  1124  3470  **3309** | 99  132  212  4  12  16  197  158  325  14  13  24  9  9  16  287  819  **768** |
| PsycINFO (Ovid) | **S1)** ((compassion* or kindness or empath* or sympath* or genero*) adj3 (unit* or department* or organisation* or organization* or communit* or practice* or design* or ward* or workplace* or culture* or core* or value* or environment* or institute* or philosop* or principle* or climate*)) and (health* or hospi*).mp. [mp=title, abstract, heading word, table of contents, key concepts, original title, tests & measures, mesh word]  **S2)** (compassion* or kindness or empath* or sympath* or genero*) adj3 (health* or hospi*).mp. [mp=title, abstract, heading word, table of contents, key concepts, original title, tests & measures, mesh word]  **S3)** (exp kindness/ or exp empathy/) and (exp hospitals/ or exp hospice/)  **S4)** S1 or S2 or S3 | 876  948  44  **1730** | 174  189  7  **344** |
| EMBASE (Ovid) | **S1)** (((compassion* or kindness or empath* or sympath* or genero*) adj3 (unit* or department* or organisation* or organization* or communit* or practice* or design* or ward* or workplace* or culture* or core* or value* or environment* or institute* or philosop* or principle* or climate*)) and (health* or hospi*)).mp. [mp=title, abstract, heading word, drug trade name, original title, device manufacturer, drug manufacturer, device trade name, keyword heading word, floating subheading word, candidate term word]  **S2)** ((compassion* or kindness or empath* or sympath* or genero*) adj3 (health* or hospi*)).mp. [mp=title, abstract, heading word, drug trade name, original title, device manufacturer, drug manufacturer, device trade name, keyword heading word, floating subheading word, candidate term word]  **S3)** (exp kindness/ or exp empathy/) and (exp hospitals/ or exp hospice/ or exp health care facility/)  **S4)** S1 or S2 or S3 | 1723  1825  2322  **5517** | 382  410  409  **1094** |
| ProQuest Dissertations & Theses Global | **S1)** noft(((Compassion* OR kindness OR empath* OR sympath* OR genero*) N/3 (unit* OR department* OR organisation* OR organization* OR communit* OR practice* OR design* OR ward* OR workplace* OR culture* OR core* OR value* OR environment* OR institut* OR philosop* OR principle* OR climate*)) AND (hospi* OR health*) )  **S2)** noft((Compassion* OR kindness OR empath* OR sympath* OR genero*) N/3 (hospi* OR health*))  **S3)** (MAINSUBJECT.EXACT("Kindness") OR MAINSUBJECT.EXACT("Empathy") OR MAINSUBJECT.EXACT("Sympathy")) AND (MAINSUBJECT.EXACT("Health facilities") OR MAINSUBJECT.EXACT("Hospitals"))  **S4)** S1 OR S2 OR S3 | 611  479  9  **1033** | 100  81  0  **166** |
