# Supplementary material for: What is the State of Organisational Compassion‐Based Interventions Targeting to Improve Health Professionals' Well‐Being? Results of a Systematic Review
Source: J Adv Nurs. 2024 Oct 7;81(5):2246–76. doi: 10.1111/jan.16484 (PMC11967289; doi:10.1111/jan.16484)
Supplement: Supplementary file 2 — Supporting Information 2. [file JAN-81-2246-s001.docx]

| ID |  |
| --- | --- |
| Study no. |  |
| Authors |  |
| Year |  |
| Journal |  |
| Study design |  |

**Supplementary File 2 – Data extraction template**

| **Design and methods** | **Paragraphs from the text** | **Pages** |
| --- | --- | --- |
| Aim of the study |  |  |
| How is the study related to compassion? |  |  |
| Study population |  |  |
| Inclusion criteria of participants |  |  |
| Exclusion criteria of participants |  |  |
| Settings |  |  |

| **Intervention** | **Paragraphs from the text** | **Pages** |
| --- | --- | --- |
| What is the intervention about? *(including how it is delivered)* |  |  |
| Duration of the intervention *(from start to last follow-up)* |  |  |
| Number of participants in the intervention and who are they? *If more than one group, describe both* |  |  |
| Any dropouts during the intervention? *(how many, who, and the reasons) If more than one group, describe both* |  |  |

| **Evaluation/Outcomes** | **Paragraphs from the text** | **Pages** |
| --- | --- | --- |
| How do they evaluate the intervention? *(e.g. pre-post-surveys, focus group interviews, etc.)* |  |  |
| Who are the respondents/informants and how many? *(if pre-post measures, describe both)* |  |  |
| Outcome measures relevant to the organisational/cultural components *(e.g. retention, turnover, teamwork, etc.)* |  |  |
| Results relevant to organisational/cultural components *(if more than one group, describe both)* |  |  |
| Do they question their own results (strengths/limitations)? *Describe it* |  |  |
| Do they question the method(s) used (strengths/limitations)? *Describe it* |  |  |
| Other notes (if needed) |  |  |
